# Integrative analysis of the molecular mechanisms, immunological features and immunotherapy response of ferroptosis regulators across 33 cancer types

**DOI:** 10.7150/ijbs.64654

**Published:** 2022-01-01

**Authors:** Bufu Tang, Ruochen Yan, Jinyu Zhu, Shimiao Cheng, Chunli Kong, Weiqian Chen, Shiji Fang, Yajie Wang, Yang Yang, Rongfang Qiu, Chenying Lu, Jiansong Ji

**Affiliations:** 1 Key Laboratory of Imaging Diagnosis and Minimally Invasive Intervention Research, Lishui Hospital, School of Medicine, Zhejiang University, Lishui 323000, China.; 2 Department of Radiology, Sir Run Run Shaw Hospital, Zhejiang University School of Medicine, Hangzhou 310016, China.; 3 Department of Radiology, Second Affiliated Hospital, School of Medicine, Zhejiang University, Hangzhou, China.; 4 School of Medicine, Zhejiang University, Hangzhou 310012, China.; 5 Department of Radiology, the Fifth Affiliated Hospital of Wenzhou Medical University, Lishui 323000, China.

## Abstract

Ferroptosis is a recently described mode of cell death caused by the accumulation of intracellular iron and lipid reactive oxygen species (ROS), which play critical roles in tumorigenesis and cancer progression. However, the underlying molecular mechanisms and promising biomarkers of ferroptosis among cancers remain to be elucidated. In this study, 30 ferroptosis regulators in ferroptosis-related signaling pathways were identified and analyzed in 33 cancer types. We found transcriptomic aberrations and evaluated the prognostic value of ferroptosis regulators across 33 cancer types. Then, we predicted and validated potential transcription factors (including E2F7, KLF5 and FOXM1) and therapeutic drugs (such as cyclophosphamide, vinblastine, and gefitinib) that target ferroptosis regulators in cancer. Moreover, we explored the molecular mechanisms of ferroptosis and found that signaling pathways such as the IL-1 and IL-2 pathways are closely associated with ferroptosis. Additionally, we found that ferroptosis regulators have a close relationship with immunity-related parameters, including the immune score, immune cell infiltration level, and immune checkpoint protein level. Finally, we determined a ferroptosis score using GSVA method. We found that the ferroptosis score effectively predicted ferroptotic cell death in tumor samples. And ferroptosis score is served as an independent prognostic indicator for the incidence and recurrence of cancers. More importantly, patients with high ferroptosis scores received greater benefit from immunotherapy. We aslo created an online webserver based on the nomogram prognostic model to predict the survival in immunotherapy cohort. The reason for this outcome is partially the result of patients with a high ferroptosis rate also having high immune scores, HLA-related gene expression and immune checkpoint protein expression, such as PDL2 and TIM3. Moreover, patients with high ferroptosis scores exhibited CD8 T cell and TIL infiltration and immune-related signaling pathway enrichment. In summary, we systematically summarize the molecular characteristics, clinical relevance and immune features of ferroptosis across cancers and show that the ferroptosis score can be used as a prognostic factor and for the evaluation of immunotherapy effects.

## Introduction

Ferroptosis has been widely researched since 2012, when Dr. Brent R. Stockwell first described erastin induction. The iron chelator deferoxamine (DFO) and the lipid reactive oxygen species (ROS) scavenger ferrostatin-1 were found to inhibit erastin induction 1. As a critical form of cell death, ferroptosis leads to oxidative damage due to excessive accumulation of iron ion-dependent lipid peroxidation products, which is exacerbated by several mechanisms, including the transport of iron ions and the production and elimination of lipid ROS. In addition, the decomposition of ferritin, the synthesis of coenzyme Q10 (CoQ10) and the catabolism of glutamine are also contributors to the ferroptosis system and affect cancer cell sensitivity to ferroptosis inducers 2, 3.

Iron is an essential nutrient for cell proliferation and growth and is involved in the occurrence and growth of cancer, the regulation of the cancer microenvironment and the metastasis of cancer cells 4. In view of its key role in cancer, iron-induced cell death has become a powerful target for cancer treatment 5. Some anticancer drugs approved by the US Food and Drug Administration (FDA) have been identified as inducers of ferroptosis 6-9. For example, ferumoxytol, an iron oxide nanoparticle that can be used to treat leukemia, reduces ferroportin levels. Therefore, predicting and validating ferroptosis inducers based on their underlying mechanisms of action and ferroptosis regulators may lead to a promising therapeutic strategy for cancer.

In the tumor microenvironment (TME), activated immune cells can secrete proinflammatory chemokines and cytokines, such as tumor necrosis factor (TNF), interleukin 6 (IL-6), IL-1β and prostaglandin E_2_ (PGE_2_), thereby promoting the proliferation of tumor cells 10, 11. Recent studies have shown that ferroptotic cancer cells may regulate tumor immunity in different ways. It has been previously demonstrated that ferroptotic cells can induce the attraction and activation of innate immune cells such as neutrophils, triggering ferroptotic cells ingestion by macrophages 12, 13. Ferroptotic cells can respond to PGE_2_ and inducible GPX4 depletion 14, 15 and release eicosanoids, such as 5-hydroxyeicosatetraenoic acid (5-HETE), 12-HETE and 15-HETE, and then interrupt the proinflammatory process driven by NF-κB pathway activation in cells stimulated with TNF or IL-1β 16. However, the various types of ferroptotic cancer cells described above may activate or inhibit different immune cells; thus, the proportion of different ferroptosis-related molecules in cancer cells, such as glutathione peroxidase 4 (GPX4), long-chain family member 4 (ACSL4), and solute carrier family 7 member 11 (SLC7A11), is a double-edged sword 3, 5, 17. It is urgent to explore the mutual regulatory mechanisms between ferroptosis-related signaling pathways and the tumor immune microenvironment (TIM).

Because the dual effects of ferroptosis on tumor immunity and immunotherapy differ from person to person, we identified 30 ferroptosis regulators based on the ferroptosis KEGG pathway and existing basic studies, and then, we classified these regulators into the ferroptosis driver group and the ferroptosis suppressor group. We then identified transcriptome-level features and the clinical relevance of the ferroptosis regulators across cancers. We performed a series of analyses to determine the molecular mechanisms, immunogenic features and clinical relevance of ferroptosis. This process of discovery involved the exploration of the relationship between ferroptosis regulators and patient survival rates, differences in ferroptosis gene expression in different cancers, and the relationship between these regulators and cancer hallmark-related pathways. We also explored whether ferroptosis regulators are associated with tumor microenvironment immune cell infiltration and whether immunotherapy efforts vary in cancers with different ferroptosis phenotypes. Therefore, a ferroptosis score was calculated for each cancer, and its relationship with the patient's prognosis score, tumor cell infiltration score, and immune checkpoint level (to evaluate tumor immunity) was determined. Moreover, we assessed how the ferroptosis score was associated with immunotherapy effects using survival model and nomogram, with an online webserver based on the proposed nomogram, and we found that the immunotherapeutic effects in patients with different ferroptosis scores differed considerably.

## Materials and Methods

### Identification of 30 ferroptosis regulators and acquisition of data

We identified 41 genes from the ferroptosis pathway map in the Kyoto Encyclopedia of Genes and Genomes (KEGG, https://www.genome.jp/kegg/pathway.html) database. After determining the attributes of the identified genes with the FerrDb database (http://www.zhounan.org/ferrdb) 18 and searching for them manually in the PubMed and Google Scholar databases to clarify their function in ferroptosis, 30 genes were classified into two groups: ferroptosis drivers or suppressors. Any ferroptosis gene that could not be definitively identified as a ferroptosis promoter or inhibitor, either because of insufficient research data or reports of dual functionality, was excluded from the study. Gene expression information and related clinical data for 33 types of cancer were downloaded from The Cancer Genome Atlas (TCGA, http://cancergenome.nih.gov/). Data on an immunotherapy cohort, including the SKCM_DFCI_2015 cohort, was downloaded from the cBioPortal database (http://www.cbioportal.org/). The GSE104462 and GSE147625 microarray datasets containing normal, control and experimental samples were obtained from the Gene Expression Omnibus (GEO) database (https://www.ncbi.nlm.nih.gov/geo/). A total of 318 tumor-related transcription factors (TFs) were obtained from the Cistrome Cancer database (http://cistrome.org/CistromeCancer/) 19. Fifty cancer hallmarks were obtained through GSEA (http://www.gsea-msigdb.org/gsea/) 20.

### Prediction of transcription factors associated with ferroptosis regulators in pan-cancer tissue

To study the relationship between tumor-related TFs and ferroptosis regulator expression in pan-cancer, we obtained data on cancer-related transcription factors from the Cistrome Cancer database (http://cistrome.org/CistromeCancer/) and then built a network based on a co-expression analysis of the TCGA pan-cancer cohort data. We screened 318 cancer-related factors one by one and confirmed that they were transcription factors. For regulators expression in pan-cancer, we standardized between different cancer types 21. The correlation coefficient standards were cor > 0.25 and *P* < 0.05. A sankey diagram was used to visualize the TF-ferroptosis regulatory network.

### Protein-protein interaction (PPI) network analysis

A PPI network was constructed based on data obtained from BioGRID (http://www.thebiogrid.org), an online database of physical and genetic interactions 22, and then processed with Cytoscape software. We chose the key PPI network modules through the molecular complexity detection (MCODE) plugin 23.

### Gene set variation analysis (GSVA)

Gene set variation analysis (GSVA) is a nonparametric unsupervised analysis method 24 that can be used to identify specific gene cohort scores of every set. We downloaded gene sets from the Molecular Signatures Database (http://software.broad-institute.org/gsea/msigdb) and then used GSVA package in R language to ascribe signaling pathway variation scores to the gene sets to evaluate their biological functions. Additionally, we performed this algorithm to distinguish high and low ferroptosis regulator expression levels and identify the relationship between ferroptosis and 50 cancer hallmark pathways.

### Functional annotation

After identifying differentially expressed genes using the limma package in R software (version 3.5.1) 25, based on |log2-fold change| > 1 and adjusted *P* < 0.05, Gene Ontology (GO) and KEGG pathway enrichment analyses were performed using the Database for Annotation, Visualization and Integrated Discovery (DAVID, https://david.ncifcrf.gov), the OmicsBean database (www.omicsbean.cn/) and the Metascape database (http://metascape.org/). An adjusted *P* < 0.05 was considered the cutoff criterion.

### Exploration of potential drug and ferroptosis targets

The data of thirty-six predicted ferroptosis drug targets and their RNA composite expression were obtained from the CellMiner database (https://discover.nci.nih.gov/cellminer/home.do). We used the average z score of compound activity (DTP NCI-60) to do relationship analysis with the RNA composite expression of ferroptosis regulators. Target genes of cyclophosphamide were analyzed by using the SwissTargetPrediction (http://www.swisstargetprediction.ch/) database and the Comparative Toxicogenomics Database (CTD, https://ctdbase.org/), both of which can predict protein targets of small molecules, to find targets identified with data from both sources 26.

### Semiflexible docking of drugs and ferroptosis regulators

We used AutoDock Vina software 27 to conduct semiflexible docking to study the interaction between cyclophosphamide and ferroptosis regulators such as GPX4. The structure of cyclophosphamide was downloaded from the PubChem database (https://pubchem.ncbi.nlm.nih.gov/) with transcoding using Open Babel 28, and the 3D structure information of the GPX4 protein was acquired from the protein database PDB (https://www.rcsb.org). During the docking analysis, cyclophosphamide was considered flexible, while the protein was rigid. When using AutoDock Vina for docking, the entire surface of the protein was considered the binding target of GPX4. Finally, we visualized model docking with PyMOL 29.

### Evaluation of the ferroptosis score

We calculated the ferroptosis score for each gene set of 30 ferroptosis regulators by utilizing the GSVA algorithm as described above. One of the input files was the gene set of 30 ferroptosis regulators, the other was the standardized gene expression matrix of patients' examples. The ferroptosis score across cancer types has been standardized. We used the X-tile program 30 to calculate the optimal cutoff value for the ferroptosis score. High tumor ferroptosis scores were above the cutoff value, and low tumor ferroptosis scores were below the cutoff value.

### Estimation of immune cell infiltration levels

To ensure the reliability of the results, we used three methods to assess the level of immune cell infiltration in a variety of cancers. First, Cell-type Identification by Estimating Relative Subsets of RNA Transcripts (CIBERSORT) analysis was performed to quantitatively convert tumor tissue transcriptome data into the absolute abundance of immune cells and stromal cells by estimating the relative subset of RNA transcripts for the evaluation of 22 tumor-infiltrating immune cells (TIICs). Standard annotation files were used to organize gene expression characteristics. The R package “CIBERSORT” was used to convert the mRNA data and detect TIICs in the tumor microenvironment. Second, the Tumor Immune Estimation Resource (TIMER, https://cistrome.shinyapps.io/timer/), a method for characterizing gene expression associations, was used to interactively study the relationship between immune infiltration (6 TIIC subsets: CD4 T cells, CD8 T cells, B cells, macrophages, neutrophils, and dendritic cells) and associated gene expression 31. TIMER employed the statistical method previously published by Li B et al. 32, using gene expression profiles to infer the abundance of TIIC. We predicted the correlation between expression of pan-cancer ferroptosis regulators and gene markers of tumor-infiltrating immune cells in correlation modules. The expression scatter plots of user-defined genes and immune cell gene markers were generated in the end, including Spearman correlation and statistical significance, which we converted into heatmap presentation. Finally, single-sample gene set enrichment analysis (ssGSEA) was employed to quantify the infiltration level of 16 immune cell markers in each SKCM patient sample on the basis of the available expression data 33. The normalized enrichment score (NES) obtained with the ssGSEA was regarded as the infiltration level of each immune cell marker. The ComBat function was used to eliminate the potential batching effect between NESs in different queues.

### Gene set enrichment analysis (GSEA)

We used a gene set enrichment analysis (GSEA) approach (http://www.broadinstitute.org/gsea/index.jsp) to determine how immune-related pathways differed in the high and low ferroptosis groups. A normalized *P*-value < 0.05 was considered to represent statistical significance.

### Survival analysis

Each mRNA was normalized by log2 transformation for further analysis. R language software was used for the data analysis and data visualization. Hazard ratio analysis was performed on 30 ferroptosis regulators, and the forestplot package was used to display the hazard ratios of the ferroptosis regulators in different cancers. Then, we compared the overall survival (OS) and progression-free survival (PFS) of patients with pan-cancer stratified by ferroptosis score. The optimal cutoff value of the ferroptosis score was determined by X-tile software. The Kaplan-Meier (KM) curve was used for this comparison, and log-rank test calculation results with a *P* < 0.05 were considered statistically significant. The hazard functions of the high and low ferroptosis score group were plotted using cubic splines.

We used the R package “survivalROC” to perform the time-dependent ROC curve analysis and calculate the area under the ROC curve (AUC) to evaluate the prognostic performance of the ferroptosis score model. The “pheatmap” package was used to visualize the correlation between different ferroptosis scores and different clinical factors.

### Establishment and evaluation of the nomogram, construction of an online webserver

Nomogram can integrate a variety of different variables that affect prognosis at the same time to obtain the survival prediction model of the studied cohort 34. Univariate and multivariate cox regression analysis were performed on clinical variables, and variables with P<0.05 were identified as independent risk factors, including age, gender, and ferroptosis score. We used Rms R software to build an OS predictive nomogram based on the above variables 35. Calibration curves were used to evaluate the calibration of the nomogram, which shows the consistency between the predicted survival rate and the actual survival results. The calibration curve is the comparison between actual risk and predicted risk. The curve is closer to the diagonal (45°), indicating that the prediction effect is better 36. We implemented the proposed nomogram in a user-friendly online webserver (https://zjuresearch.shinyapps.io/dynnomapp/), which is convenient for use by clinicians and researchers.

### Identification of ImmuneScore, StromalScore, and ESTIMATEScore in tumor tissue

To assess tumor immunoactivity, we estimated the abundance of stromal cells and immune cells based on 'Estimation of Stromal and Immune cells in malignant tumors using Expression' (ESTIMATE, https://sourceforge.net/projects/estimateproject/) data, a method that calculates the proportion of immune and stromal cells in each tumor sample, thereby quantifying tumor purity (level of immune cell invasion) based on the expression of immune genes in the sample 37. In detail, we performed the ESTIMATE algorithm in R language to estimate the proportion of immune or stromal components in the TME, where we got the following scores: ImmuneScore, StromalScore, and ESTIMATEScore, which are positively associated with the ratio of immune components, stromal components, and the sum of both components. The higher the scores are, the larger the ratio of the corresponding components in the TME.

### Determination of TMB and MSI

To explore the relationship between ferroptosis and TMB and MSI in pan-cancer tissues, the MSI status and tumor mutation burden of pan-cancer samples were obtained from the cBioPortal database (https://www.cbioportal.org/).

### Statistical analysis

mRNA level was normalized by log2 transformation for further analysis. R language was used for data analysis and data visualization. The differences between the groups were compared by Wilcox test. The Spearman method was used to calculate the correlation between two variables. *P* < 0.05 was considered to be statistically significant. Continuous variables are expressed as the mean ± standard deviation (SD), and the differences are expressed by t-test results, while categorized variables are expressed as rates or numbers. Log-rank test was used to compare survival time differences.

## Results

### Identification of transcriptomic aberrations and the prognostic role of ferroptosis regulators across 33 cancer types

**Figure 1:**
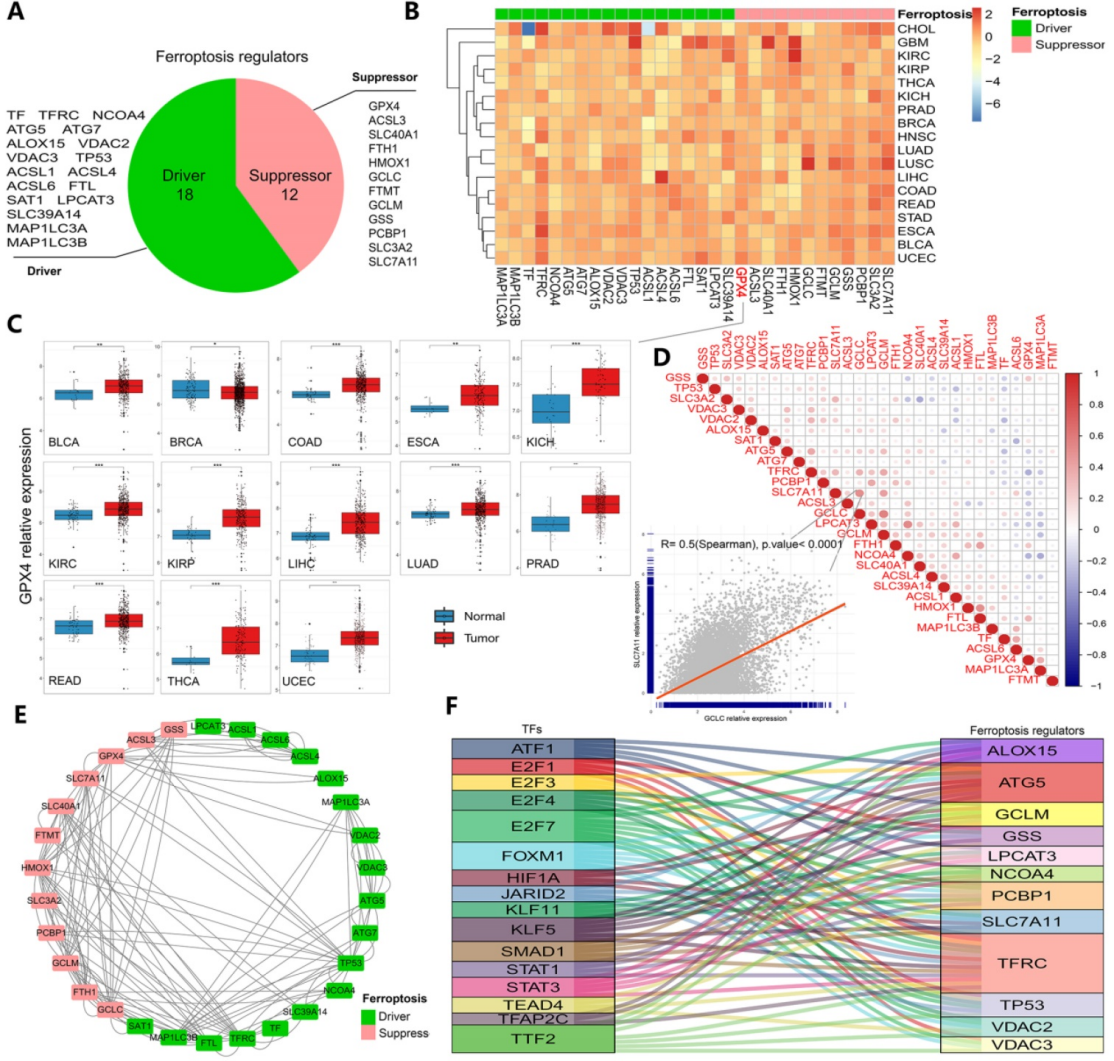
** Transcriptomic aberrations of ferroptosis regulators across 33 cancer types. (A)** The variation in 30 ferroptosis regulators. Red shows suppressors, and green shows drivers. **(B)** Heat map showing the expression of ferroptosis regulators across 33 cancer types. Red indicates upregulation, and blue indicates downregulation. The expression in each cancer type has been normalized (normal tissue vs. tumor tissue). **(C)** Box plots of GPX4 expression across 13 cancer types. *P-value < .05, **P-value < .01, ***P-value < .001. **(D)** Diagrams of the correlations between the expression levels of ferroptosis regulators at the pan-cancer level. The scatter plot represents the correlation between GCLC and SLC7A11. **(E)** Protein-protein interaction network (PPI network). Red represents suppressors, and green represents drivers. The line indicates the interaction between two proteins. **(F)** Sankey diagram showing the association between ferroptosis-related transcription factors and ferroptosis regulators. The relation lines showed in figure all have statistically significance.

The whole research process is represented by a flowchart in Figure S1. First, after summarizing ferroptosis regulators by KEGG pathway enrichment and selecting genes using the FerrDb database, PubMed and Google Scholar, 30 genes were categorized into two groups: ferroptosis drivers and ferroptosis suppressors (Figure 1A). To determine ferroptosis-related gene expression across cancer types, 33 cancer type datasets covering more than 11000 samples were obtained from the TCGA database (Table S1). The results showed that certain genes (such as GPX4, SLC7A11 and GSS) displayed higher expression, while others (such as ACSL1 and ACSL4) displayed lower expression in some cancer types (Figures 1B-C and S2A-E). In particular, GPX4 expression, which is shown in Figure 1C, was significantly increased in 13 cancers (all at *P* < 0.05, Figure 1C). GPX4 is a key enzyme in ferroptosis, and erastin consumes glutathione, thereby inhibiting the GPX enzyme system. RSL3 also triggers ferroptosis by binding to GPX4 15, 38. Moreover, the hazard ratios of every regulator were determined in 33 cancer types, and we found that the majority of the regulators exhibited comparable associations with patient survival (Figures S2 and S3, Table S2). TFRC (a ferroptosis driver) was found to be a powerful risk factor for 9 cancers, namely, ACC, BLCA, CESC, KICH, KIRP, LGG, LIHC, PAAD and THCA. GPX4 was found to be a protective factor against BRCA, CESC, THCA and UCEC (Figure S2). To further clarify the transcriptome characteristics of ferroptosis regulators, correlation diagrams and protein-protein interaction networks were generated (Figure 1D and E). Among the identified genes, the expression of SLC7A11 exhibited a strong relationship with GCLC expression (*P*<0.0001, Figure 1D). Protein-protein interactions were prevalent among 30 ferroptosis regulators, especially SLC7A11 and HMOX1 (drivers) and MAP1 LC3B and TFRC (suppressors). These results indicate differences in the expression of ferroptosis regulators across cancers and differences between expression patterns and patient prognosis, suggesting important roles for ferroptosis regulators in different cancers.

### Determination and validation of transcription factors and therapeutic agents targeting ferroptosis regulators in cancer

**Figure 2:**
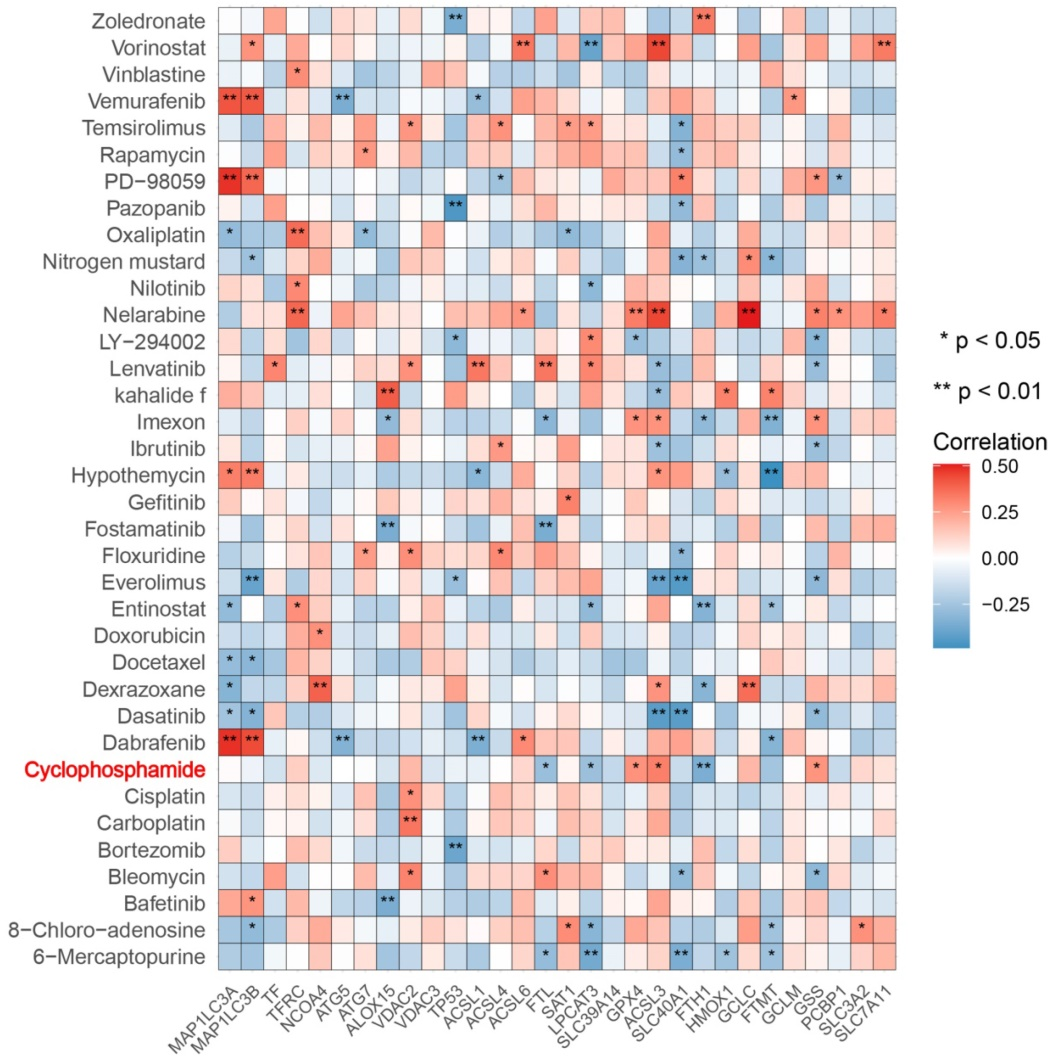
** Correlation between 36 predicted ferroptosis-targeted medicinal compound activities and the expression of ferroptosis regulators.** Red represents a positive correlation, and blue represents a negative correlation. *P-value < 0.05, **P-value < 0.01, ***P-value < 0.001.

**Figure 3:**
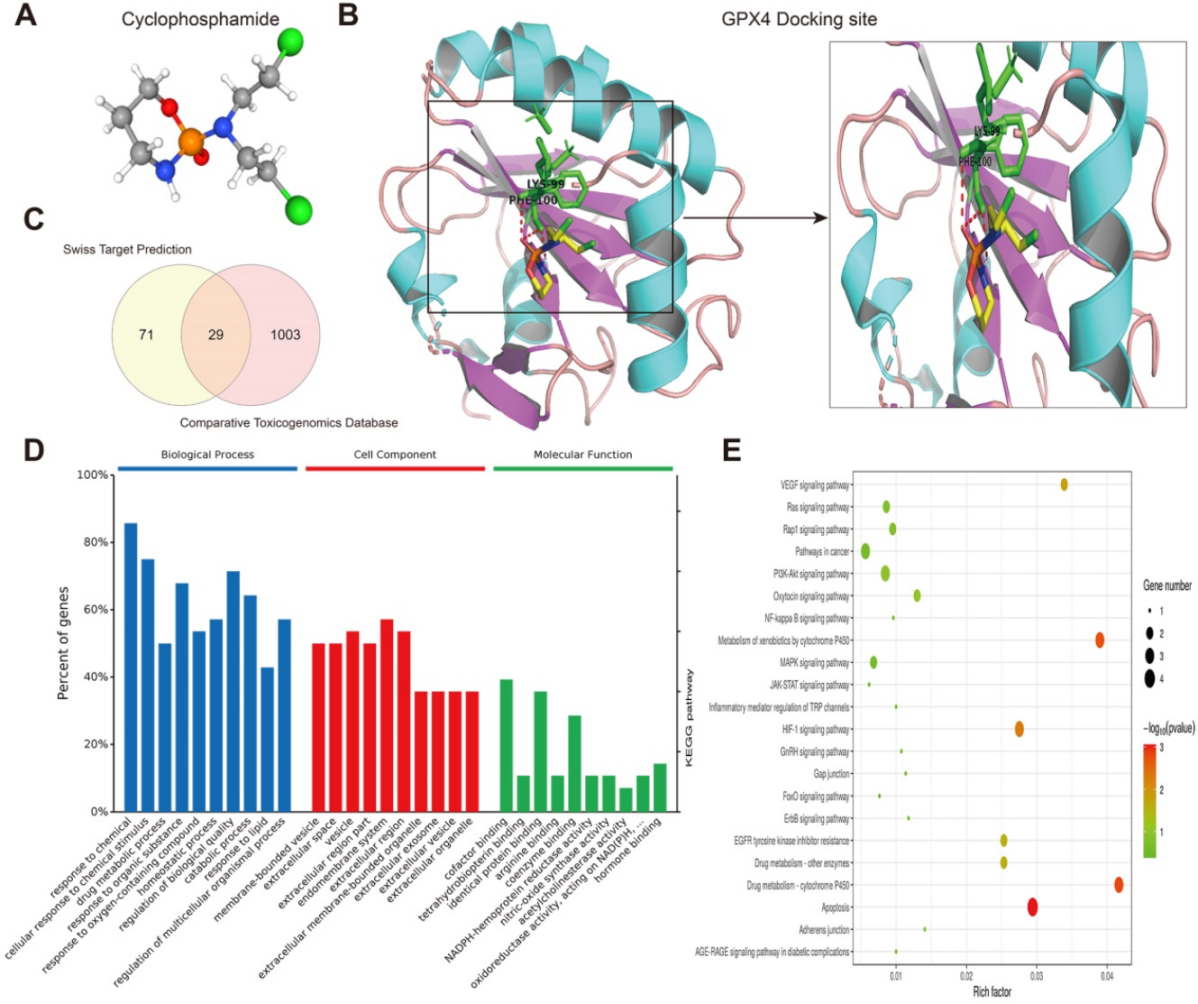
** Prediction of therapeutic agents for use in ferroptosis regulation. (A)** The molecular structure of cyclophosphamide. **(B)** Diagram of cyclophosphamide and protein combination.** (C)** Genes targeted by cyclophosphamide were predicted by the Swiss Target Prediction and Comparative Toxicogenomics Database. **(D)** Gene Ontology (GO) enrichment analysis of 1103 targeted genes, including those in biological process, cell component and molecular function categories. All percent of the genes shown were 10 most enriched cell signaling pathways. **(E)** The KEGG enrichment analysis of the 1103 targeted genes.

Next, we predicted transcription factors using the Cistrome database to analyze the greatest number of direct upstream transcription factors among the ferroptosis regulators. The Sankey diagram showed that some ferroptosis regulators were significantly correlated with upstream transcription factors (Figure 1F). We also explored the average z score of therapeutic drug (DTP NCI-60) activity related to the RNA composite expression of ferroptosis regulators and predicted 36 potential ferroptosis agents (including cyclophosphamide), and the connection between ferroptosis-related gene expression levels and 36 predicted ferroptosis agents was demonstrated (Figure 2). Cyclophosphamide is an immunosuppressant and an anticancer drug that selectively targets cancer cells 39 and is widely used as a chemotherapy drug 40. Figure 3A shows the three-dimensional structure of cyclophosphamide, and Figure 3B shows the molecular docking sites of the cyclophosphamide and GPX4 proteins. As shown in the figure, lysine at position 99 and isoleucine at position 100 of GPX4 can form a noncovalent bond with cyclophosphamide (Figure 3B). The binding energy obtained from the docking of GPX4 with cyclophosphamide was -3.91 kcal/mol on Autodock Vina. Since cyclophosphamide can bind to the GPX4 protein, it is a potential ferroptosis inducer (Figure 3B). We also observed possible targets of cyclophosphamide by using the Swiss Target Prediction and Comparative Toxicogenomics Database (Figure 3C). In addition, Gene Ontology (GO) and KEGG enrichment analyses of 29 targets common to the results obtained from both databases are shown (Figure 3D and E). These genes were mostly enriched in the biological process category, particularly “homeostatic process”, “NF-kB signaling pathway”, and “JAK-STAT signaling pathway” (Figure 3D and E). The results indicate that these upstream transcription factors and therapeutic drugs of ferroptosis regulators, especially cyclophosphamide, may be regarded as ferroptosis inducers as they target the ferroptosis suppressor GPX4.

### Exploration of the molecular mechanism of ferroptosis in the erastin-treated and GPX4-knockdown cohorts and in cancer hallmark-related pathways

**Figure 4:**
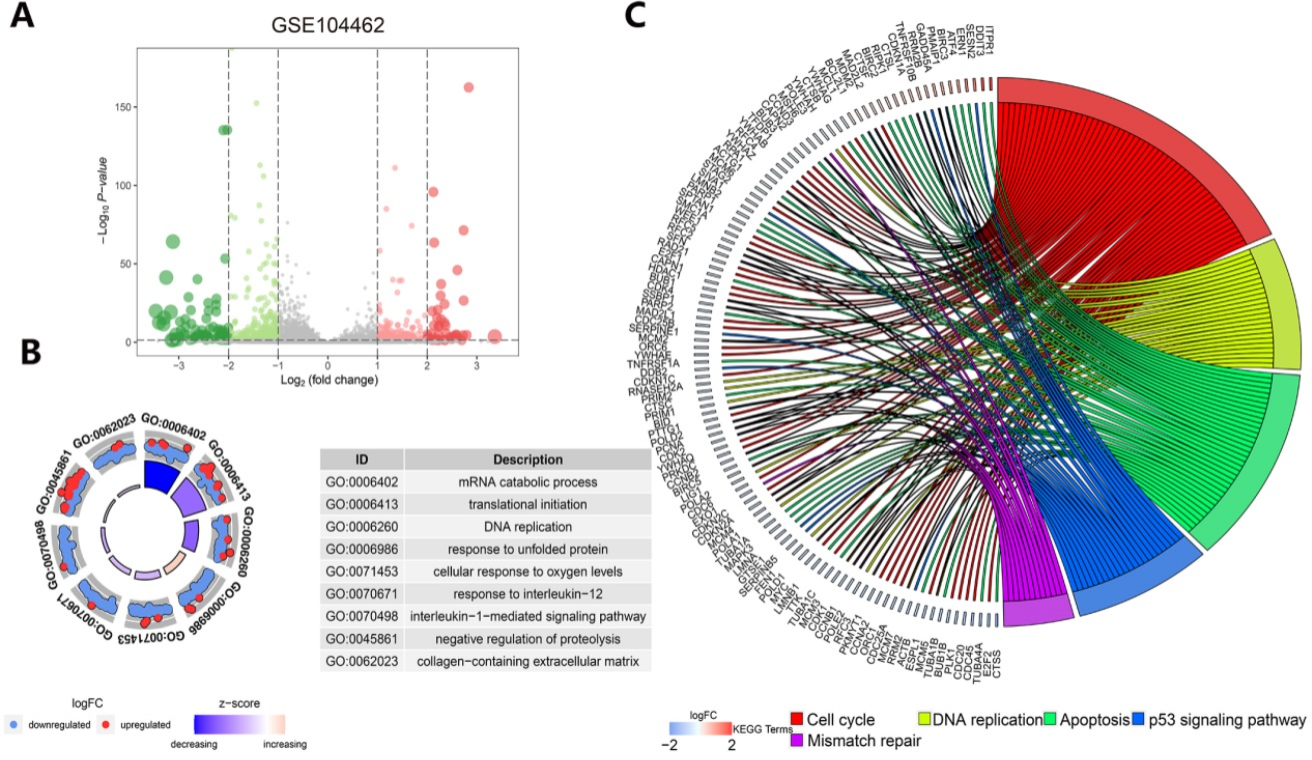
** Functional and pathway enrichment analysis in liver cancer cells after erastin treatment. (A)** The expression distribution of genes in the GSE104462 dataset. The size of the circles represents the degree of |log2-fold change|. **(B)** GO enrichment analysis of ferroptosis regulators. **(C)** KEGG enrichment analysis of ferroptosis regulators in 4 biological processes.

**Figure 5:**
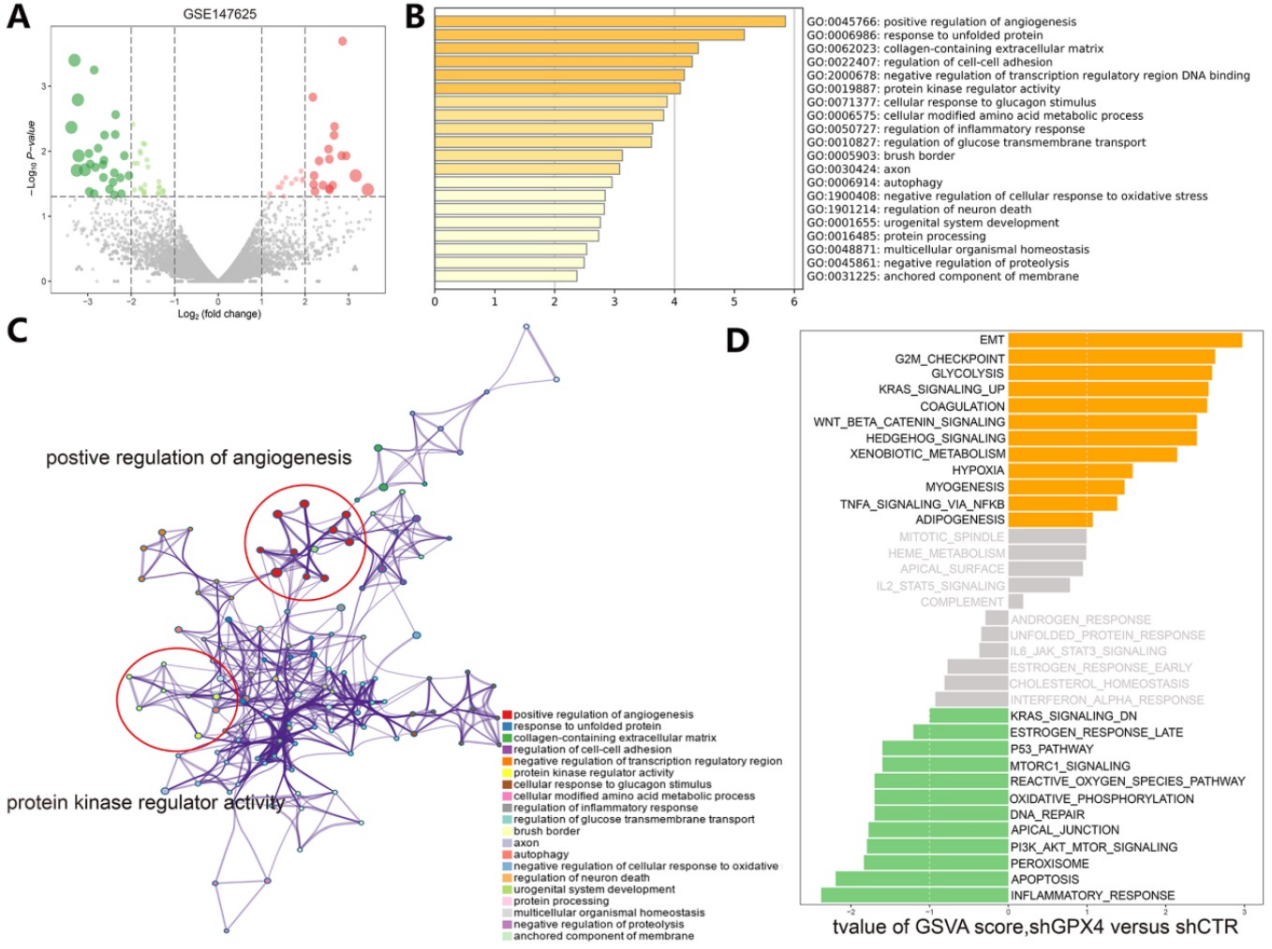
** Functional and pathway enrichment analyses and GSVA-enrichment analysis of GPX4-knockdown liver cancer cells compared to normal cells. (A)** The gene expression distribution in the GSE147625 dataset. **(B)** GO enrichment analysis of gene expression in GPX4-knockdown cells. **(C)** KEGG enrichment analysis of ferroptosis regulators in GPX4-knockdown cells. **(D)** The bar plots indicate the distribution of the t values of the GSVA scores calculated for several pathways.

Understanding the crosstalk between ferroptosis regulators is of great importance; thus, we first investigated differentially expressed genes (DEGs) in the GSE104462 cohort (liver cancer cells with or without erastin treatment) (Figure 4A). Erastin is an inducer of ferroptosis, as previously described 1. After erastin treatment, ferroptosis regulators were up- or downregulated in liver cancer cells, and we could identify the biological processes of these ferroptosis regulators. Hence, GO functional enrichment analysis and KEGG pathway analysis were performed with the goal of understanding the functions of the differentially expressed ferroptosis genes in tumor immunization, and we found that pathways (IL-1-mediated signaling) and biological processes (response to IL-12, DNA replication and apoptosis) were highly enriched in the erastin treatment cohort (Figure 4B-C). To further explore the molecular mechanism of ferroptosis, we also used the GSE147625 cohort (wild-type and GPX4-knockdown trophoblasts). Figure 5A shows the DEGs identified in the GSE147625 cohort. In addition, biological processes, including positive regulation of angiogenesis and protein kinase regulator activity, were enriched (Figure 5B and C). The GSVA analysis indicated that signaling pathways such as the G2/M checkpoint and TNF-α signaling via NF-kB had highly differential pathway scores, suggesting their positive relationship with ferroptosis-related processes.

**Figure 6:**
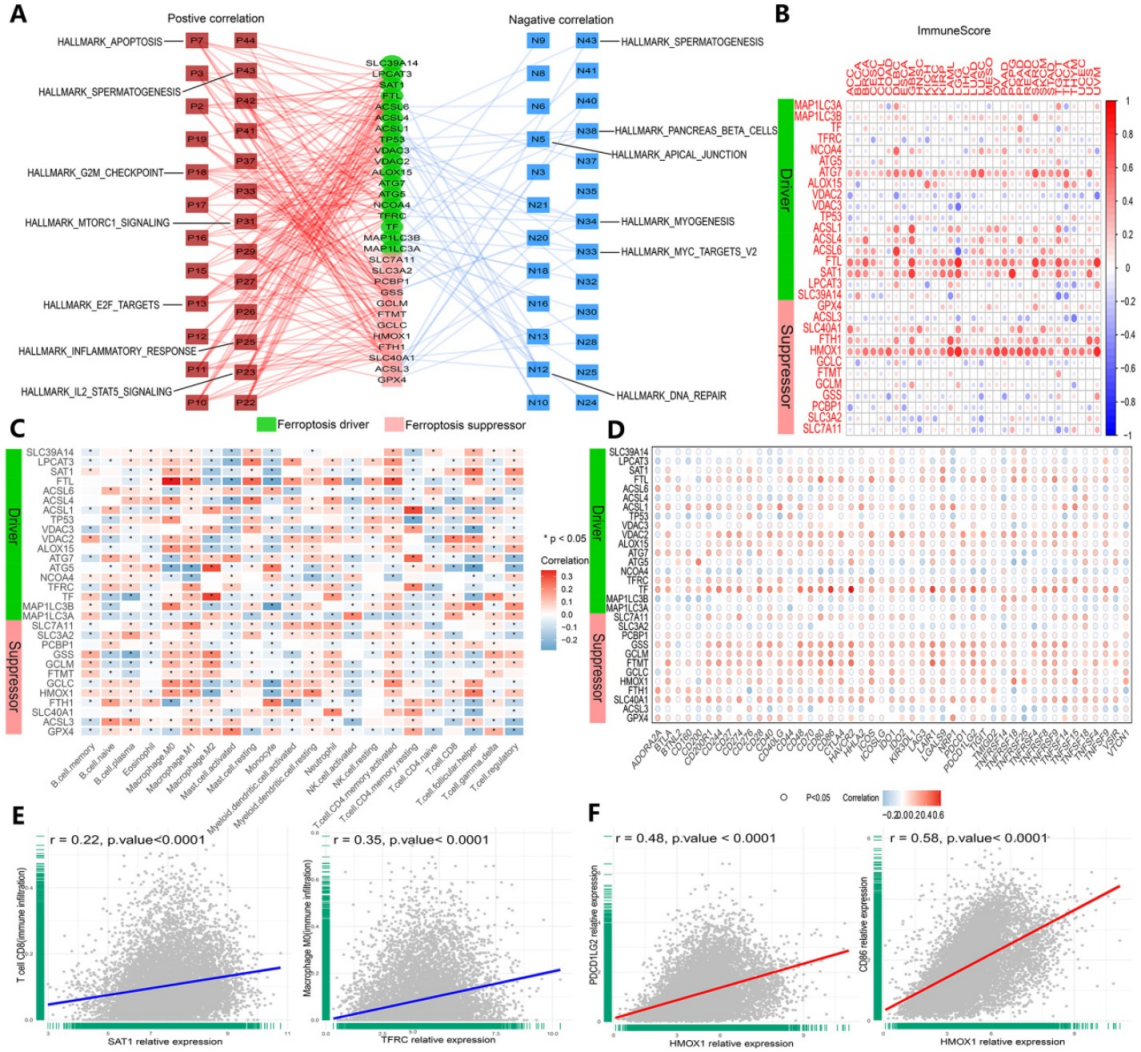
** Ferroptosis regulators are affiliated with cancer hallmark-related pathways and tumor immunity. (A)** Network planning of the correlation between ferroptosis regulation and 50 cancer hallmark-related pathways. On both sides, red indicates positive, and green indicates negative. Intermediately, ferroptosis drivers are shown in red, and suppressors are shown in blue. **(B)** Correlation analysis between ferroptosis regulator expression and ImmuneScore across 33 cancer types. **(C)** The correlation of ferroptosis regulator expression with the infiltration levels of 22 immune cells. *P-value < .05, **P-value < .01. **(D)** The correlation of ferroptosis regulator expression with 47 immune checkpoints. **(E)** The scatter plots show the correlation between CD8+ T cells and activated memory CD4+ T cells and SAT1 and TFRC. **(F)** The scatter plots represent the extent to which HOMX1 expression is related to PDCD1 LG2 and CD86 expression.

To further understand how ferroptosis regulators affect cancer, we inspected the relationship between ferroptosis regulators and 50 cancer hallmark-related pathways (Figure 6A, Table S3). The results showed that TP53, FTL and SLC40A1 were positively correlated with the inflammatory response and IL2/STAT5 signaling pathways. In contrast, ALOX15, TP53 and SLC40A1 were negatively correlated with MYC targets and DNA repair pathways (Figure 6A). These approaches were used to explore the molecular mechanisms of ferroptosis, and the results validated the close association between ferroptosis regulators and cancer signaling pathways.

### Evaluation of the association of ferroptosis regulators and immunogenic features in cancer

We validated the association between ferroptosis regulators and immunogenic characteristics among cancers. First, we evaluated the relationship between ferroptosis regulators and stromal and immune scores among cancers. Stromal and immune scores have been proven to be related to the occurrence of colorectal cancer and affect the clinical prognosis 41. The ESTIMATE algorithm can be used to predict the proportion of stromal cells and immune cells infiltrating tumor tissue 37. Based on this calculation, the ESTIMATE scores obtained in this study were found to be positively correlated with both types of cells, and tumor purity was estimated on the basis of this score combination. Therefore, to expand our evaluation of ferroptosis regulator correlations with tumor immunogenicity, we calculated the immune score, stromal score, ESTIMATE score and tumor purity of 33 cancer types, and the correlation of scores with the expression of 30 genes was measured (Figures 6B and S4A-C). The expression levels of the HMOX1, FTL, SAT1 and ATG7 genes were highly correlated with the aforementioned indicators in each cancer dataset (Figures 6B and S4A-D). Then, we determined the relationship between ferroptosis regulator expression levels and immune cell infiltration levels by CIBERSORT (Figure 6C and E), and similar results were shown with the TIMER algorithm (Figure S4D). For GPX4, a marked positive correlation with macrophages (M1 and M2), activated mast cells, and resting memory CD4+ T cells was found, and a negative correlation with M0 macrophages, resting mast cells, follicular T cells and regulatory T cells (Tregs) was found (Figure 6C). SAT1 and TFRC were strongly and positively correlated with CD8+ T cells and CD4+ memory T cells, respectively (Figure 2E). Subsequently, we investigated the interaction between the expression of 47 common immune checkpoint genes and the levels of ferroptosis regulators (Figure 6D and F). For example, the expression of HMOX1 was closely and positively correlated with PDCD1 LG2 and CD86 expression levels (Figure 6F). Intriguingly, these results confirmed that ferroptosis regulators play critical roles in tumor immunity.

### Identifying the prognostic value of the ferroptosis score associated with clinical relevance across 33 cancer types

**Figure 7:**
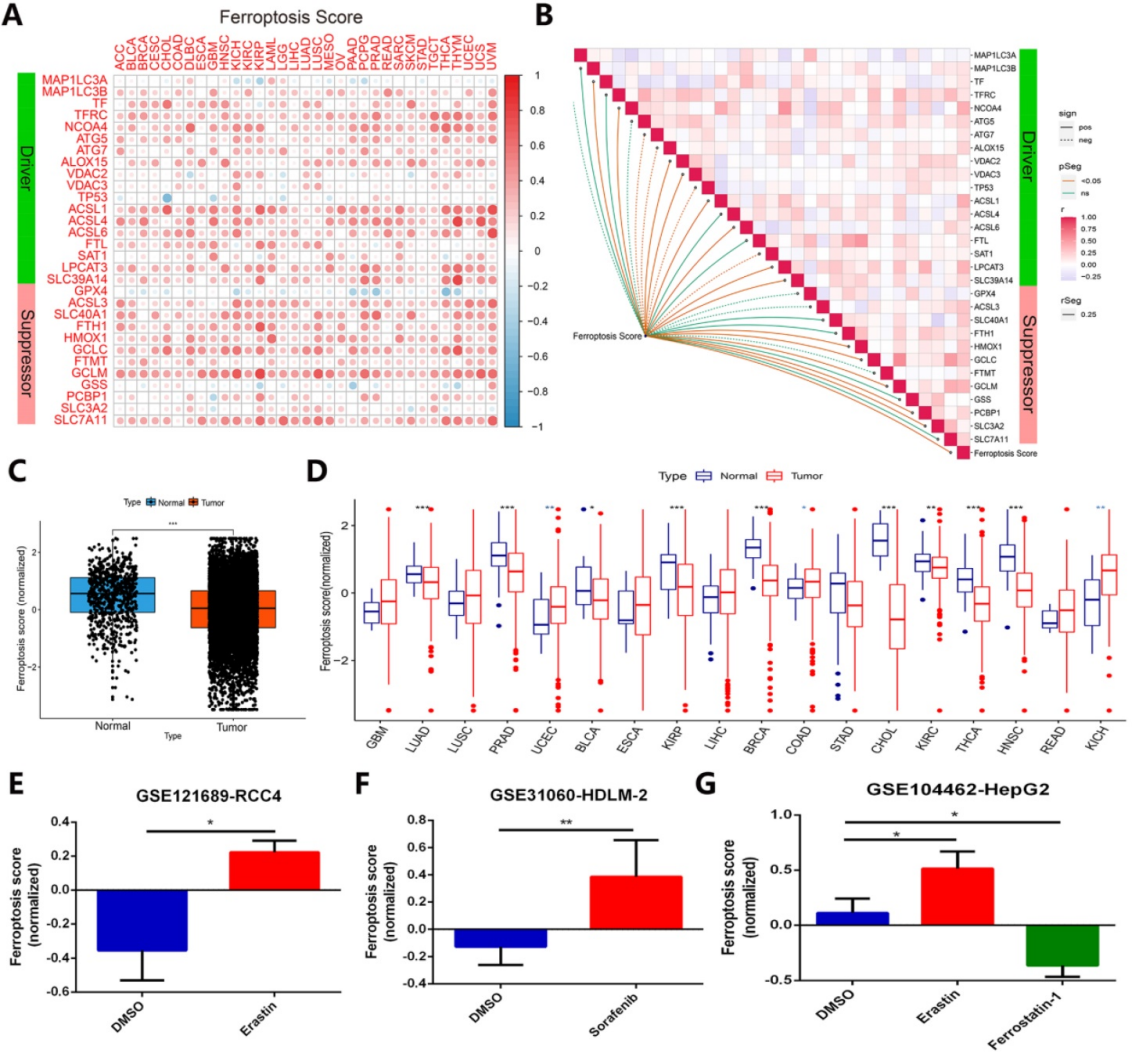
** Ferroptosis score can exhibit ferroptosis status across cancer types effectively. (A)** The relationship between ferroptosis score of 33 cancer types and ferroptosis expression. **(B)** Diagram of the correlation between ferroptosis score in pan-cancer and ferroptosis regulators expression. **(C)** Average value of ferroptosis score in normal tissue and all (11000+) tumor samples. **(D)** Ferroptosis score in normal tissue and tumor tissue of comparable cancer types. **(E-G)** Ferroptosis score comparison under ferroptosis inducers (erastin and sorafenib) or inhibitor (ferrostatin-1), datasets are GSE121689, GSE31060 and GSE104462.

To identify whether ferroptosis regulators contribute to clinical risk prediction, we first calculated the ferroptosis score for each gene set by utilizing gene set variation analysis (GSVA) 24. In order to verify whether the ferroptosis score calculated by GSVA can predict the ferroptosis status in cancer cells in different cancer types or pan-cancer, we have done the following verification. A relationship of ferroptosis score in different cancer type with ferroptosis regulators expression was presented in Figure 7A. Most of the ferroptosis regulators showed a good positive correlation with the ferroptosis score of different cancer types, except for individual genes such as TP53, GPX4 and GSS (Figure 7A). Figure 7B displayed the correlation between ferroptosis score of all samples in TCGA and regulators expression in pan-cancer. In TCGA pan-cancer samples, the ferroptosis scores of more than 11,000 cancer samples were compared with normal tissues, and it was found that the overall state of ferroptosis was down-regulated in tumor samples (Figure 7C). Among the imparity between normal tissue and tumor tissue, ferroptosis status of most comparable cancer types is down-regulated, such as LUAD, PRAD, BLCA, KIRP, BRCA, CHOL, KIRC, THCA, HNSC (Figure 7D). Most of these cancer types have been proven to inhibit the growth of cancer cells by activating ferroptosis in previous studies 42-46. In addition, we verified ferroptosis score changes of cancer samples under treatments of ferroptosis agonists and inhibitors in the three data sets, respectively GSE121689, GSE31060, and GSE104462 (Figure 7E-G). The results displayed that ferroptosis scores are up-regulated under treatment of two types of ferroptosis stimulants, erastin and sorafenib, but down-regulated using ferroptosis inhibitor ferrostatin-1, in line with the activation or inhibition of cancer cells ferroptosis status 15, 47, 48. Combining with the above, ferroptosis score can effectively represent the ferroptosis status in cancer cells.

**Figure 8:**
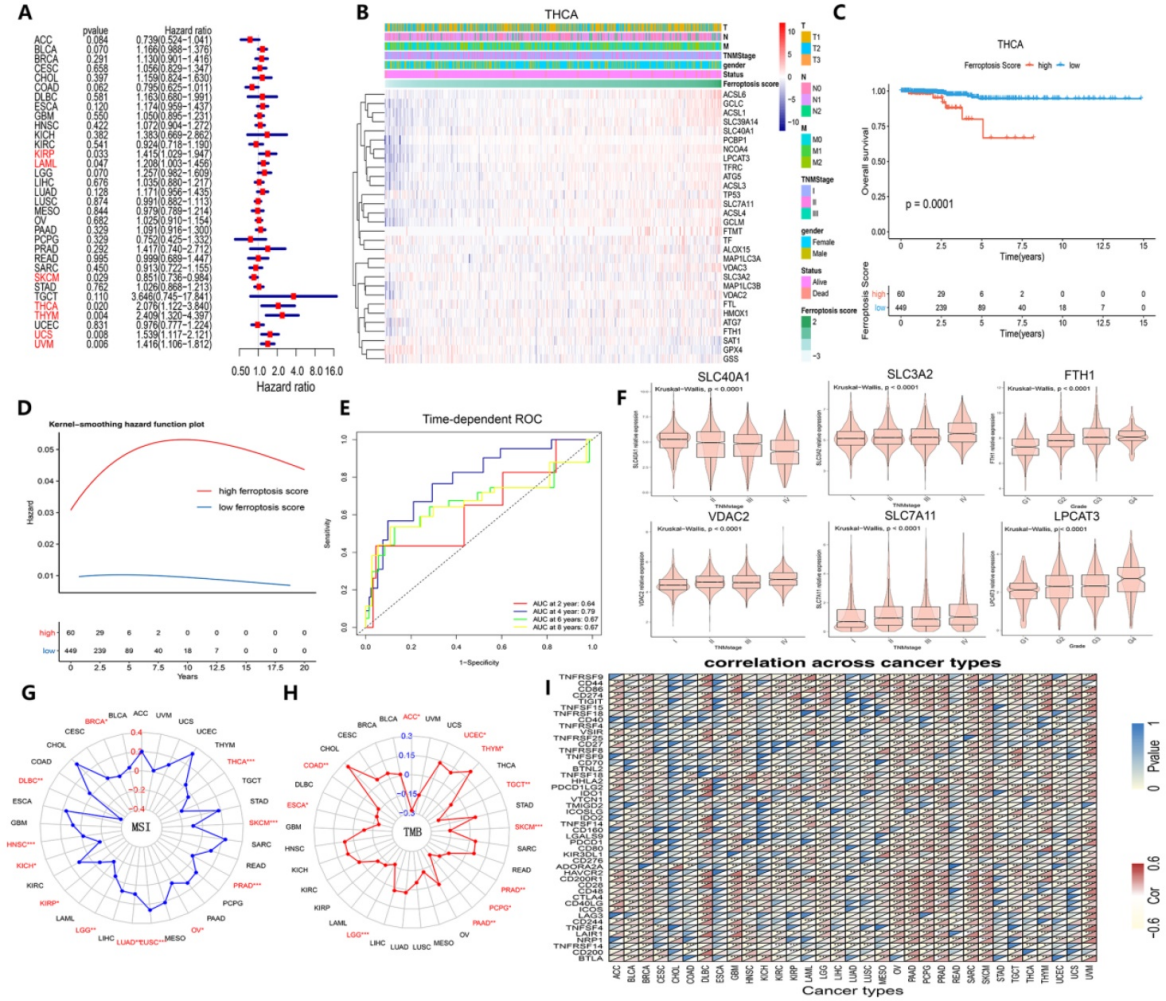
** Identification of the clinical role of the ferroptosis score across cancers. (A)** The distribution diagram (forest plots) of hazard ratios across dozens of cancer types. The cancer types whose hazard ratio was greater than 1 and *P* value was less than 0.05 are colored red. **(B)** Heat map of clinicopathologic features in THCA patients based on the expression of ferroptosis regulators. **(C)** The Kaplan-Meier overall survival (OS) curve of two clusters distinguished by ferroptosis score. **(D)** Kernel-smoothing hazard function plot of the high and low ferroptosis groups. **(E)** Time-dependent ROC curves of the 2-, 4-, 6-, and 8-year models of patients with THCA. **(F)** The violin plot indicates the TNM stage of SLC40A1, SLC3A2, FTH1, and VDAC2 and pathological grade of SLC7A11 and LPCAT3 in pan-cancer tissues. **(G-H)** The relationship between MSI/TMB and ferroptosis score in pan-cancer tissues. **(I)** Correlation between immune checkpoints and ferroptosis score across 33 cancer types.

Next, we launched analysis of the clinical significance of ferroptosis score in cancer. Using X-tile software, we categorized the samples into the following two groups: the high ferroptosis score group and the low ferroptosis score group. A higher ferroptosis score indicated a link with worse survival of patients with KIRP, LAML, THCA, THYM, UCS and UVM but better survival for patients with SKCM (Figure 8A). We further examined patients' overall survival by using K-M curve analysis, which suggested that patients with high ferroptosis scores had a substantially worse prognosis than patients with low ferroptosis scores, except for those with SKCM (Figures 8C and S5). The hazard ratios and K-M curves in the progression-free survival analysis yielded the same results (Figure S6). We validated the independent prognostic ability of the ferroptosis score between cancers, and the THCA cohort was subjected to subsequent analysis. Two groups in the THCA cohort were then identified based on their clinical characteristics (Figure 8B). The hazard ratios and the time-dependent cluster-based ROC analysis of the stratified patients with THCA supported the initial finding that the ferroptosis score may be a prognostic factor (the areas under the curve (AUCs) for the 2-, 4-, 6-, and 8-year survival rates were 0.64, 0,79, 0,67 and 0.67, respectively) (Figure 8D and E).

We then determined whether ferroptosis regulator expression was associated with clinical characteristics and found that SLC3A2, VDAC2, and SLC7A11 expression increased continuously with TNM stage and that SLC40A1 expression decreased (Figure 8F). Additionally, FTH1 and LPLCAT3 expression increased continuously with pathological grade (Figure 8F). MSI, TMB and immune checkpoints are 3 immunotherapy effectiveness indicators 49-53; thus, we assessed their association with ferroptosis scores across various cancer types to explore whether ferroptosis regulators can provide new insight into immunotherapy effectiveness (Figure 8G-I). MSI was closely correlated with ferroptosis in THCA and SKCM cells, while TMB was tightly associated with ferroptotic UCEC and TGCT cells (Figure 8G and H). Immune checkpoint gene expression also showed a prevalent association with ferroptosis score (Figure 8I). As Figure S7 demonstrates, these 6 genes were expressed differently in 6 immune subtypes 54. The ferroptosis score was higher in the inflammatory subtype, and the expression of ACSL1, NCOA4, SAT1, SLC39A14, and SLC40A1 was also higher in the inflammatory subtype. Therefore, the ferroptosis score, which is closely associated with clinical relevance, especially immunogenic features, can be used as an independent prognostic indicator among cancers associated with the immune response.

### Comparison of immunotherapy response and immunogenic features between the high ferroptosis score groups and low ferroptosis score groups in the immunotherapy cohort

**Figure 9:**
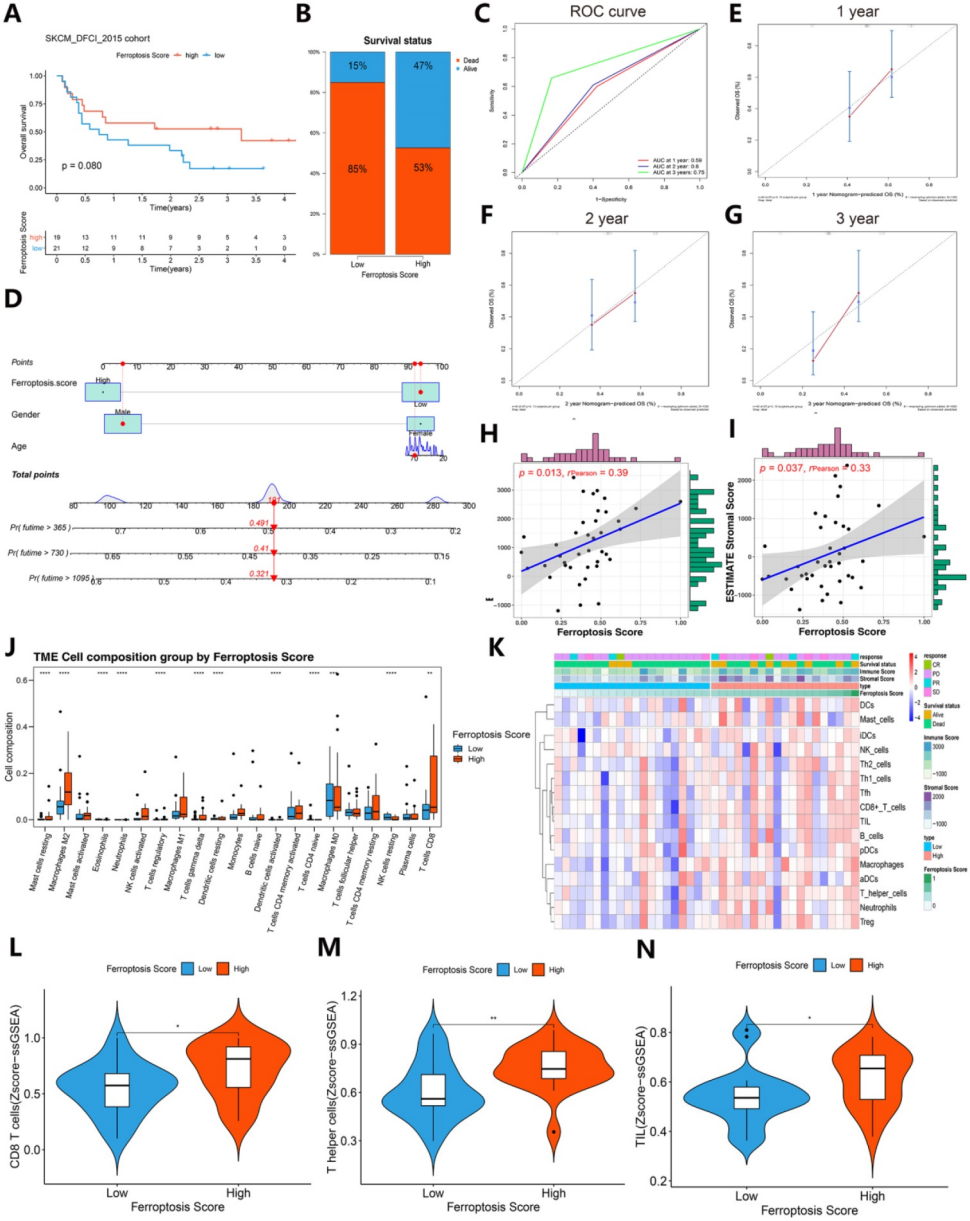
** Contrast of immunotherapy responses between the high ferroptosis score groups and low ferroptosis score groups. (A)** Kaplan-Meier survival curves showed the survival differences between the high ferroptosis score cluster and the low ferroptosis score cluster in SKCM after immunotherapy. **(B)** Survival status of patients in cohorts with different ferroptosis scores after immunotherapy. Alive status is represented by blue, and dead status is represented by red. **(C)** Time-dependent ROC curves of the 1-, 2-, and 3-year models of patients with SKCM under immunotherapy. **(D)** Nomogram of patients survival rate prediction. **(E-G)** Calibration curves of the nomogram. **(H-I)** The scatter diagram shows that the ferroptosis score was associated with the ESTIMATE stromal and immune cell scores. **(J)** Box plots of HLA gene expression grouped by ferroptosis score. **(K)** Heat map of the correlation between ferroptosis score, immunological characteristic and immune infiltration cell. **(L-N)** The violin diagram indicates the z-score of CD8+ T cells, T helper cells and tumor-infiltrating lymphocytes (TILs) depicted by high and low ferroptosis scores.

To further validate the role of the ferroptosis score in the immunotherapy response and immunogenic features, an immunotherapy cohort (SKCM DFCI 2015 cohort) was selected for subsequent evaluation. Interestingly, after immunotherapy, the survival status (85%:15% vs. 53%:47%) was largely increased in the SKCM DFCI 2015 cohort patients with high ferroptosis scores (Figure 9A-B). The ROC curves of 1-, 2-, 3-year of patients survival prognosis is 0.59, 0.6 and 0.75. A nomogram was generated based on three independent factors: age, gender and ferroptosis score, and calibration curves of 1-, 2-, 3-year of these patients OS were in accordance with reality. In addition, we built an online webserver (https://zjuresearch.shinyapps.io/dynnomapp/) based on the nomogram, which can obtain the corresponding survival plot according to the patient's age, gender and ferroptosis score, and it is convenient for clinicians to predict survival probability of immunotherapy treated patients (Figure S8).

**Figure 10:**
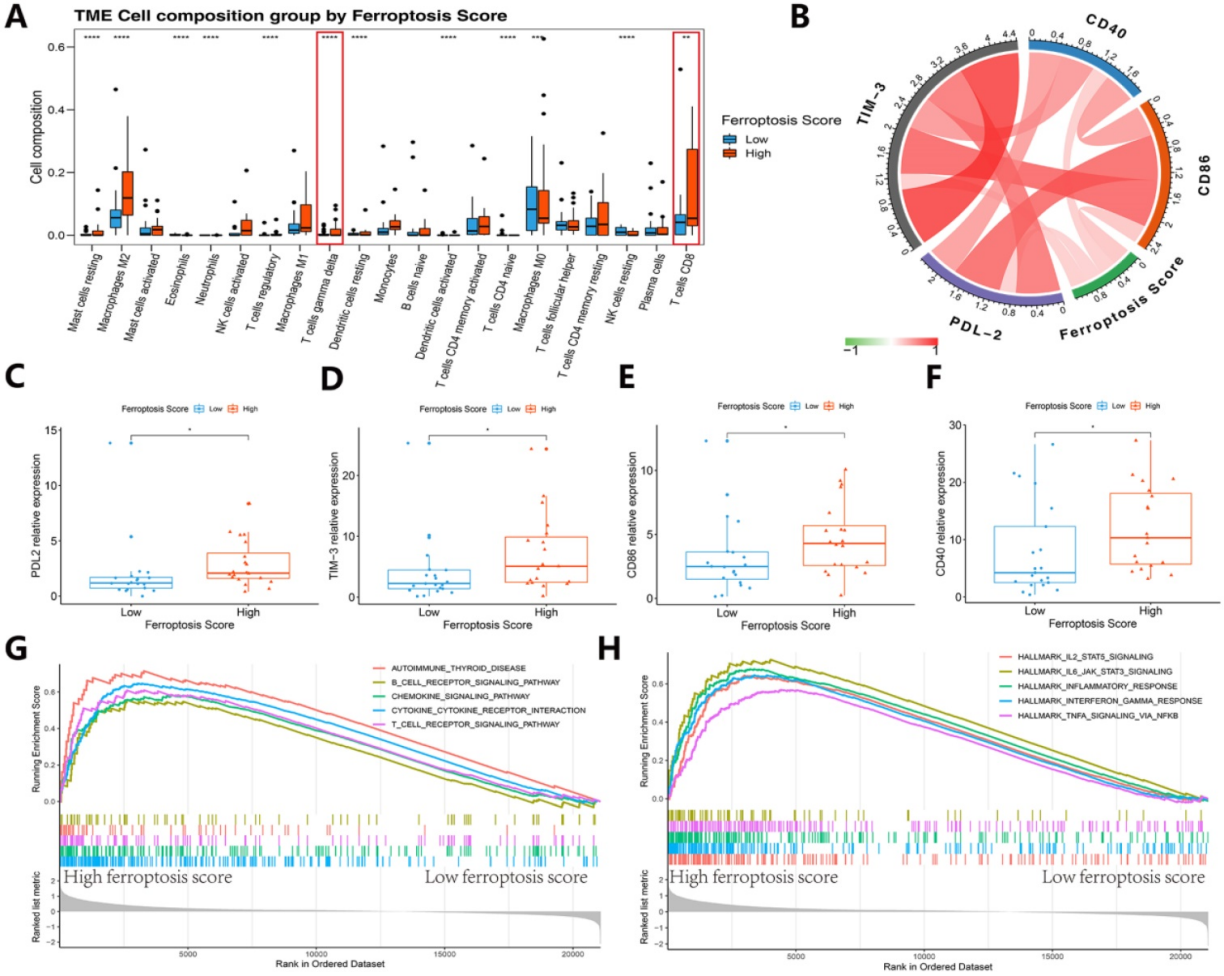
** Immunogenic features of different ferroptosis score groups in the immunotherapy cohort. (A)** Box plots of distinct TME immune cells grouped by ferroptosis score. The immune cell composition was quantified using CIBERSORT. **(B)** Association between ferroptosis score and 4 immune checkpoints. **(C-F)** The box plots indicate that there was a difference in PD-L2, TIM-3, and CD86 expression levels between clusters with high and low ferroptosis scores. **(G-H)** GSEA-enrichment plot of the 5 most enriched immune pathways and 5 most enriched cancer hallmark pathways.

A general correlation between the tumor immune microenvironment and ferroptosis score was found (Figures 9J-N and Figure 10). These results suggest that the ferroptosis score can predict the prognosis of patients with SKCM after immunotherapy and may also play a role in predicting the prognosis of patients receiving immunotherapy for other cancers.

We then refocused on the cellular and molecular levels and analyzed the relationship between the ferroptosis score and tumor immune signatures (Figures 9 and 10). The higher ferroptosis score group showed a higher immune infiltration score, HLA expression, immune cell infiltration including CD8 T cells and other TIL cells and immune checkpoint expression (Figures 9J-N and 10A-F). Additionally, the gene set enrichment analysis (GSEA) indicated that a high ferroptosis score was positively correlated with immune-related pathways, including 5 GO pathways and 5 hallmark pathways (Figure 10G-H). These results show that the ferroptosis score has a close association with the immunotherapy response and tumor immune microenvironment.

## Discussion

Ferroptosis is a newly identified form of programmed cell death, and research has gradually deepened our understanding of ferroptosis in recent years 1. However, the relationship between ferroptosis and inflammation, tumors, and immunity is not yet clear. Inducing tumor cell ferroptosis is a potential treatment strategy but might be a double-edged sword 11. Therefore, pan-cancer analyses of ferroptosis regulators were performed to attain a better understanding of the mechanisms involved in regulating ferroptosis and their impacts on the cancer immune microenvironment and to provide clinical sample data regarding the therapeutic induction of tumor cell ferroptosis. We also explored whether different levels of ferroptosis regulator expression indicate suitable targets for immunotherapy.

We first show the differential expression of 30 ferroptosis regulators in integrative cancer types. The heat map shows that ferroptosis regulator expression is substantially different between tumor samples of 18 cancers and paired normal samples, but there was no significant expression difference between drivers and suppressors. The difference in expression between cancer and normal samples suggests more functions of ferroptosis regulators in cancer. We found that the expression of GPX4 was significantly increased in 13 cancers. GPX4 is a selenoprotein, and GSH is an important cofactor 55, 56. GPX4 can reduce lipid peroxides to corresponding lipid alcohols and inhibit the occurrence of lipid peroxidation, which negatively regulates ferroptosis. A number of studies have shown that inhibiting the expression of the GPX4 gene can effectively kill tumor cells through ferroptosis. For example, it has been confirmed that the expression of the GPX4 gene is beneficial to the survival of cancer cells in a state of high mesenchymal therapy drug resistance 57. The lack of GPX4 gene expression can prevent mouse tumor recurrence, but it is necessary to determine whether GPX4 is an oncogene further.

The survival analysis shows that each ferroptosis regulator is related to the prognosis of a certain number of cancer types, while the risk factor or the protective factor depends on the situation. Research by Su Zhang et al. showed that more than one-half of iron metabolism-related genes are related to the prognosis of KIRC 58, which is consistent with our results. In addition, our results show that TFRC is a risk factor in nine cancers, but TFRC is a suppressor of ferroptosis, demonstrating that the promotion or inhibition of cancer by each ferroptosis regulator is affected by many factors.

Drugs targeting ferroptosis to treat cancer are gradually being discovered. Erastin and RSL3 are compounds that were originally found by phenotypic screening, and they have selective lethal effects on genetically engineered tumor cells. RSL3 is a GPX4 inhibitor. Inhibition of HSF1-HSPB1 pathway activity can promote erastin induction of ferroptosis in human cervical cancer cells, osteosarcoma cells and prostate cancer cells 43. Diffuse large B-cell lymphoma and renal cell carcinoma have also been found to be effectively targeted by ferroptosis inducers 59. Sorafenib can induce ferroptosis and inhibit the growth of liver cancer cells 47. Through the prediction of ferroptosis drugs and the correlation analysis between compound activity and RNA expression, we constructed a model of the interaction between GPX4 and cyclophosphamide. The functional enrichment analysis of the targets on which cyclophosphamide acts confirmed that the pathways related to these targets are processes that are affected by chemical substances.

Next, we focused on exploring the molecular mechanism of ferroptosis regulators, as well as their relationship with tumor immunity. Elastin treatment of GPX4-knockout cancer cells is strongly associated with immune pathways such as the IL-1 and IL-2 pathways. The importance of GPX4 in immune cells has been studied. Antigen-specific CD8+ and CD4+ T cells lacking GPX4 cannot spread. Knockout of GPX4 in myeloid cells can increase intestinal epithelial cell gene mutations through the accumulation of ROS and thus stimulate intestinal tumors and invasion 60, 61. GPX4 inhibitors, including RSL3 and DPI10, can directly inhibit the accumulation of ROS and lead to ferroptosis 15, but there has been no basic research on the relationship between GPX4-knockout tumor cells and immune cells. Wang et al. found that after treating mouse tumor models with PD-L1 inhibitors, the tumor volume was significantly reduced and ROS levels considerably increased 62. After the addition of the ferroptosis inhibitor lipoxstatin-1, the effect of these PD-L1 inhibitors was diminished, indicating that ferroptosis plays an important role in immunotherapy. Researchers believe that the main mechanism underlying this effect is that IFNγ released by CD8+ T cells downregulates the expression of SLC3A2 and SLC7A1 in tumor cells and inhibits the uptake of cystine by tumor cells. Our correlation results show that SCL3A2 expression is negatively correlated with the infiltration score of CD8+ T cells in tumor tissues, which provides some evidence for this hypothesis. The high correlation of HMOX1 with CD86 and PDCD1 LG2 also suggests that interactions between tumor cell ferroptosis and immune cells need to be studied in more detail. In addition to the promotion of ferroptosis by CD8+ T cell release factors, CD8+ itself can take up fatty acids through CD36, so that CD36 loses its anti-tumor effector function, and it also induces CD8+ to move toward ferroptosis and reduce intracellular cytokine production, which we thought can be a new target and explore more 63. HMOX1, or heme oxygenase (HO)-1, is a critical mediator of ferroptosis that can respond to electrophilic stimulation and is known for its anti-inflammatory effects 64. Previous studies have shown that HMOX1 is elevated in various malignant tumors, can prevent drugs from attenuating the increase in ROS, and can also promote tumor cell proliferation and metastasis 65. However, it plays different roles in diverse cancers due to drug resistance, the effects of the cancer itself or other mechanisms 66-70. Combined with the abovementioned clear correlation between HMOX1 protein interactions and immune scores, we believe that before verifying HMOX1 as a target for cancer treatment, it is necessary to explore the role of HMOX1 in the tumor immune microenvironment and ferroptosis.

We suspect that ferroptosis regulators may be independent prognostic factors in cancer. Considering previous studies, we can see that ferroptosis has been studied in hepatocellular carcinoma, breast cancer, lung cancer, pancreatic cancer, gastric cancer, cervical cancer and other tumors exhibiting ferroptosis suppression 71. In our previous study, we found that a prognostic model consisting of four genes (ABCB6, FLVCR1, SLC48A1 and SLC7A11) can effectively and independently predict the prognosis of patients with hepatocellular carcinoma 72. To determine the current expansive role of ferroptosis regulators in cancer, we calculated the ferroptosis score based on the expression of the 30 regulators in each sample set. Previous prediction model constructions were performed with single-factor Cox regression analysis to identify prognosis-related genes 73, 74; however, we first calculated the ferroptosis score and then screened related cancers. The results of the survival analysis showed that the ferroptosis score, as an independent prognostic factor, had high HR values in KRIP, LAML, SKCM, THCA, THYM, UCS and UVM. The K-M curve analysis and ROC verification of the prediction of THCA confirmed that the ferroptosis score is a satisfactory independent prognostic factor for THCA. Except for THYM (which is in the low ferroptosis group and the only sample available), the K-M curve and PFS analysis with ferroptosis considered a risk factor showed the feasibility of using the ferroptosis score for predicting the prognosis of the five other cancers. Similarly, the difference in the expression of ferroptosis regulators in TNM staging also suggests that it is related to tumor metastasis and progression. For cancers with high ferroptosis scores and poor prognosis, such as those similar to THCA, that is the clinical theoretical basis of basic research which needs more discoveries.

Since ferroptosis regulators are significantly related to the immune cell infiltration score, ferroptosis regulators may be related to the patients' immunotherapy response. Cancer immunotherapy functions a different mechanism than traditional radiotherapy, chemotherapy or targeted therapy, bringing new hope to cancer patients 75. However, due to the different immunogenicity of each type of tumor and the complex immunosuppressive mechanism in the TME, tumor immunotherapy is based on significant cancer and individual differences. Considering the characteristics of melanoma, such as extensive T cell tumor infiltration and high TMB, immunotherapy is expected to be both highly practical and effective 76. The anti-CTLA-4 antibody ipilimumab and PD-1 inhibitors nivolumab and pembrolizumab for targeting immune checkpoints have been approved for the treatment of locally advanced or metastatic melanoma 77.

In 2019, Lang et al. proved that the combination of radiotherapy and immunotherapy can induce ferroptosis, indicating that ferroptosis is at the intersection of radiotherapy and immunotherapy and is thus of great significance for tumor treatment 78. However, the impact of ferroptosis regulators on immunotherapy is not yet clear; thus, we focused on the relationship between the ferroptosis score and immunotherapy efficacy and its possible causes. Surprisingly, in the SKCM_DFCI_2015 cohort, the survival rate of the group with high ferroptosis scores compared with the group with low ferroptosis scores was nearly 32% lower after immunotherapy, and there was a significant difference in the survival analysis results, which were based on the K-M curve. Nomogram is often used to predict the prognosis of cancer patients, and it is a visual prediction tool that contains multiple variables 79. Therefore, we also visualized the survival of immunotherapy cohort with different ferroptosis scores in turn, and the predicted results were consistent with the real situation. We used the prognostic indicators of immunotherapy, TMB 49 and MSI 80 to conduct correlation analysis with ferroptosis regulators and made some interesting findings. Most ferroptosis regulators in the 33 types of cancer we analyzed were associated with MSI, TMB and immune checkpoint expression, especially in BRCA, DLBC, GBM, LGG, OV, PAAD, PCPG, PRAD, SARC, SKCM and UVM. The results of our analysis showed that patients with high ferroptosis scores in the immunotherapy cohort had poor survival status; thus, high ferroptosis scores were a risk factor in this immunotherapy cohort. This finding supported previously obtained results. After analyzing the clinical characteristics of the two groups of patients, we found that those with high ferroptosis scores had higher immune cell infiltration scores, particularly related to CD8+ T cells, T helper cells and TILs. Simultaneously, a correlation between the ferroptosis score and four types of immune checkpoints, TIM-3, CD40, CD86 and PDL-2, was also observed, and these checkpoints all exhibited higher expression in people with high ferroptosis scores. In the immune GSVA analysis, genes related to immune pathways were almost all enriched when the ferroptosis score was high. Wang's group further analyzed patients with melanoma and concluded that their prognosis was negatively correlated with the expression of SLC3A2 and positively correlated with the expression of IFNγ and CD8 62. T cell-mediated promotion of tumor ferroptosis is not only a novel mechanism for tumor immunotherapy but also a promising and powerful strategy for antitumor therapy when ferroptosis promotion treatment is combined with a checkpoint blockade. For the cancer types reported in this study, researchers should focus on the interaction between ferroptosis, inflammation and immunity in the TME. In addition, in the design of therapeutic compounds, scientists should also consider the interaction between ferroptosis and the TME.

Our research has some limitations. Pan-cancer analysis was used to evaluate the roles of ferroptosis regulators in many respects, but it is difficult to explain the mechanism of each cancer type with precision. We intend to examine the regulation of ferroptosis regulators in various cancer types, the characteristics of the immune microenvironment, and their relationship with prognosis and immunotherapy. In addition, all the data in our study were obtained from the TCGA and GEO databases, but some cancer types cannot be analyzed completely because of insufficient data (including THCA cohort which didn't receive immunotherapy) or the lack of adjacent tissue controls. What's more, it needs more datasets to verify the correspondence between ferroptosis score and ferroptosis status in cancer cells. For the online webserver construction of the nomogram, it needs more information about variables, and the selection of other treatments is ignored in the process of building prognostic nomogram. Due to space limitations, we cannot discuss each regulator in detail, and more information is needed and can be obtained through drug combination analysis.

## Conclusion

We systematically sorted and identified 30 ferroptosis regulators, comprehensively analyzed the differences in the posttranscriptional protein levels of ferroptosis regulators in different cancers, established a protein-protein expression network, and showed that ferroptosis regulators are significantly associated with cancer hallmark pathways, tumor immunity mechanisms, and tumor immune microenvironment components. Additionally, the ferroptosis score was calculated based on ferroptosis regulators by GSVA. The results indicated that the ferroptosis score is an independent prognostic marker for incidence and recurrence with superior predictive performance, and patients with higher ferroptosis scores may have a lower survival rate. Moreover, we linked ferroptosis with immunotherapy, proving that the ferroptosis score can be a potential biomarker of the immune response in the tumor microenvironment and the therapeutic response to immunotherapy.

## Supplementary Material

Supplementary figures.

Supplementary tables.

## Abbreviations

AUC: area under curve
GSEA: gene set enrichment analysis
GSVA: gene set variation analysis
HR: hazard ratio
MSI: microsatellite instability
OS: overall survival
PFS: progress free survival
ROC: receiver operating characteristic
TCGA: The Cancer Genome Atlas
TMB: tumor mutation burden
