# Supplementary material for: Integrative analysis of the molecular mechanisms, immunological features and immunotherapy response of ferroptosis regulators across 33 cancer types
Source: Int J Biol Sci. 2022 Jan 1;18(1):180–98. doi: 10.7150/ijbs.64654 (PMC8692154; doi:10.7150/ijbs.64654)
Supplement: Supplementary file 1 — Supplementary figures. [file ijbsv18p0180s1.pdf]

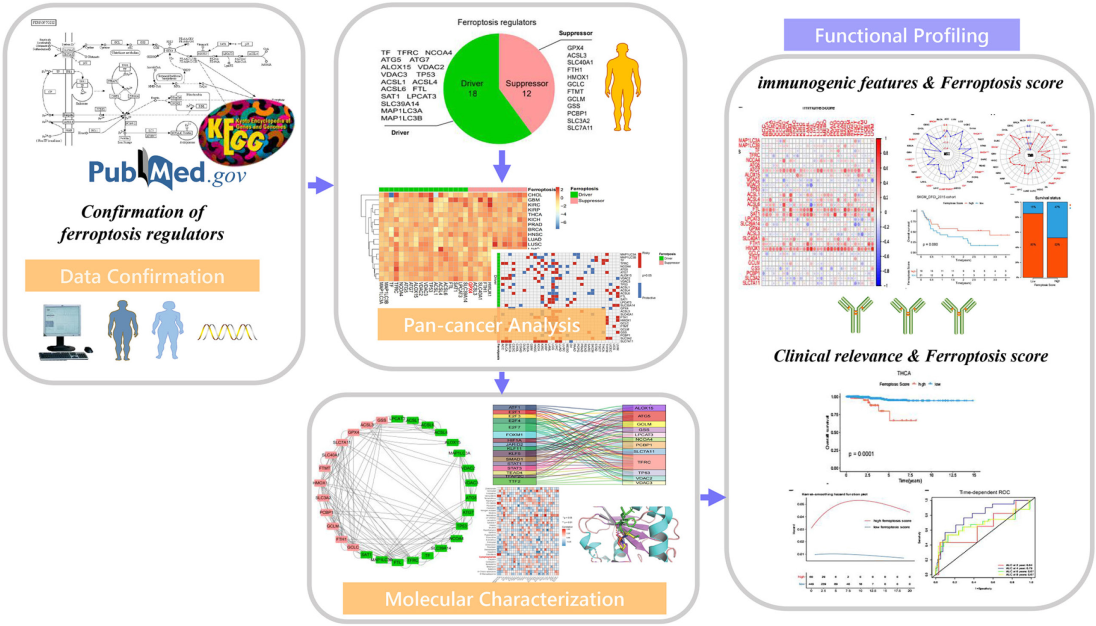

Supplementary Figure 1. Flowchart of the integrated analysis of the molecular mechanisms, immunogenic features and clinical relevance of ferroptosis regulators across 33 cancer types.

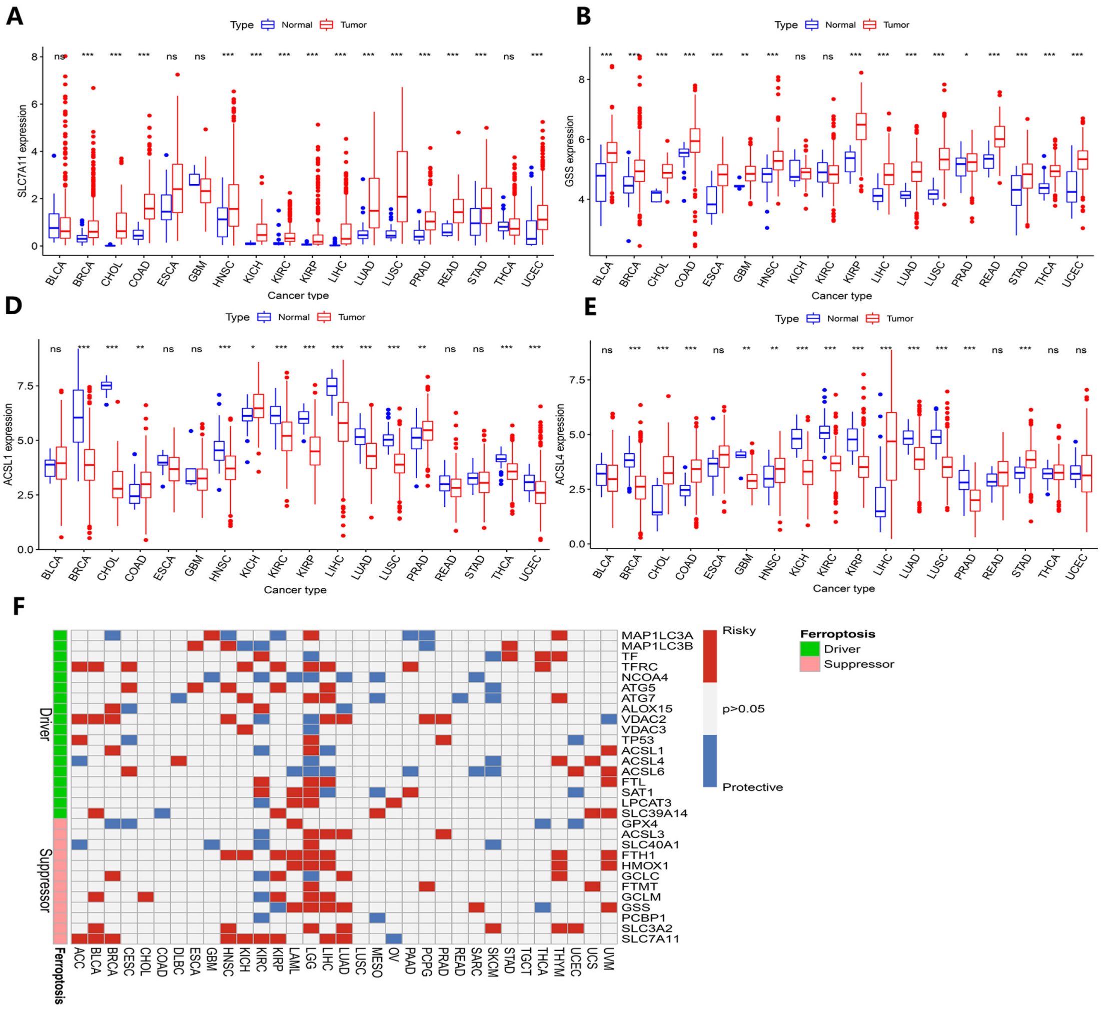

Supplementary Figure 2. Identification of transcriptomic aberrations and prognostic roles of ferroptosis regulators in pan-cancer tissue. (A-E) The box plot indicates the expression levels of ferroptosis regulators, including SLC7A11, GSS, ACSL1 and ACSL4, in pan-cancer tissue. (F) Heat map showing the association between the expression level of ferroptosis regulators and patient survival in the pan-cancer cohort. Higher expression with worse survival is represented by red, while better survival is represented by blue.

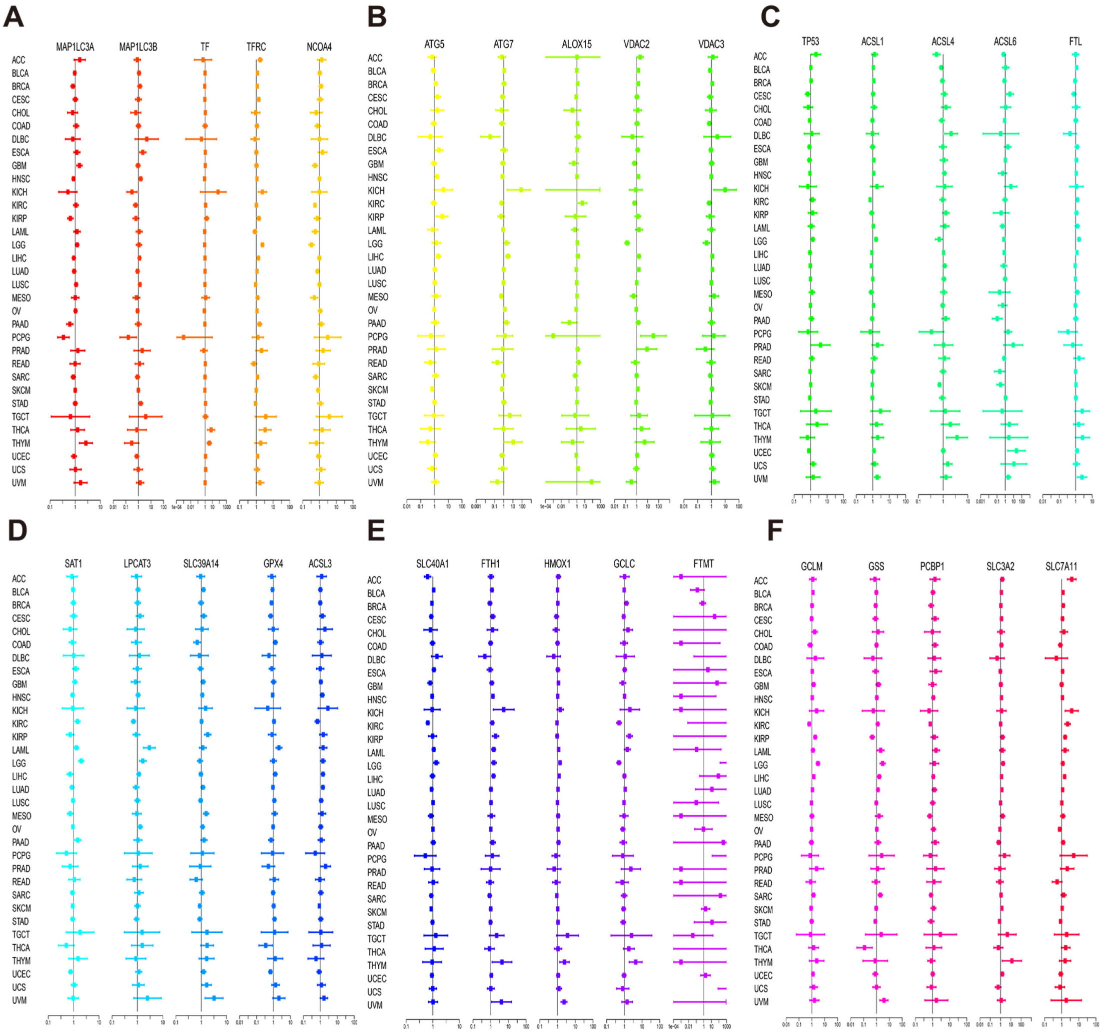

Supplementary Figure 3. The distribution of 30 ferroptosis gene hazard ratios across 33 cancer types.

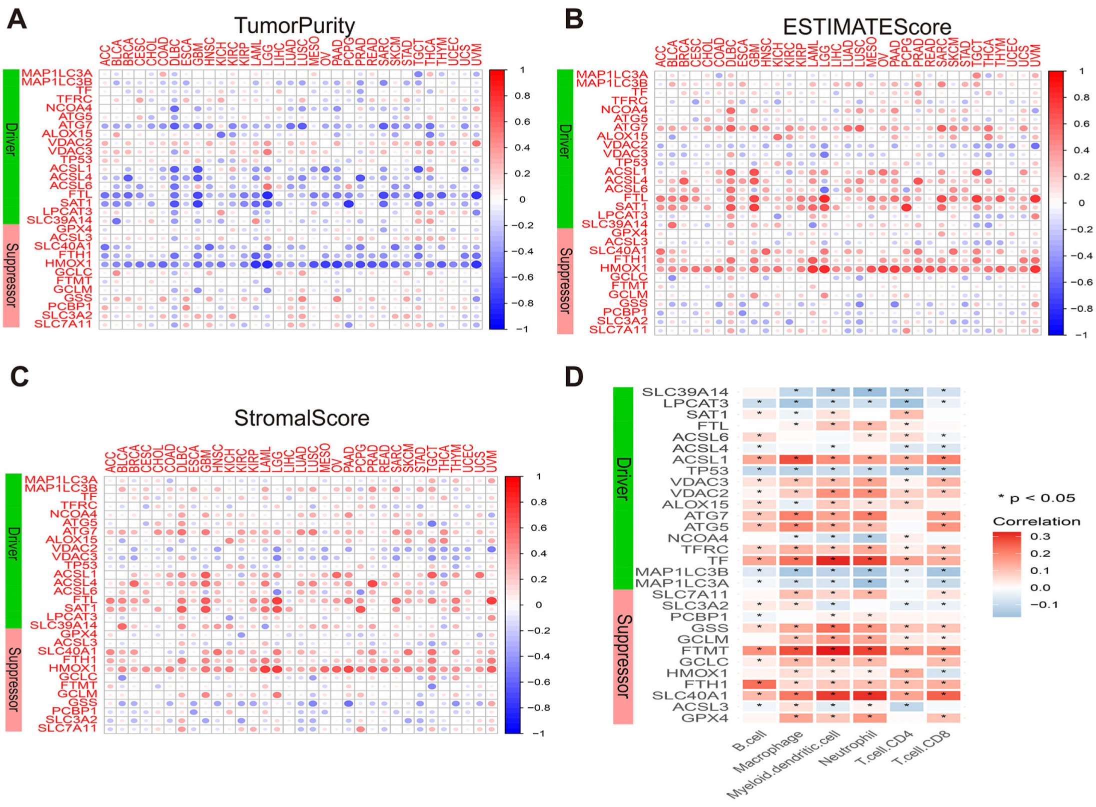

Supplementary Figure 4. Association between the expression level of 30 ferroptosis genes and tumor immunity. (A-C) The relationship between ferroptosis gene expression level and tumor purity, ESTIMATE and stromal score. **(D)** The heat map shows the association between ferroptosis gene expression levels and the abundance of 6 immune cells in the microenvironment.

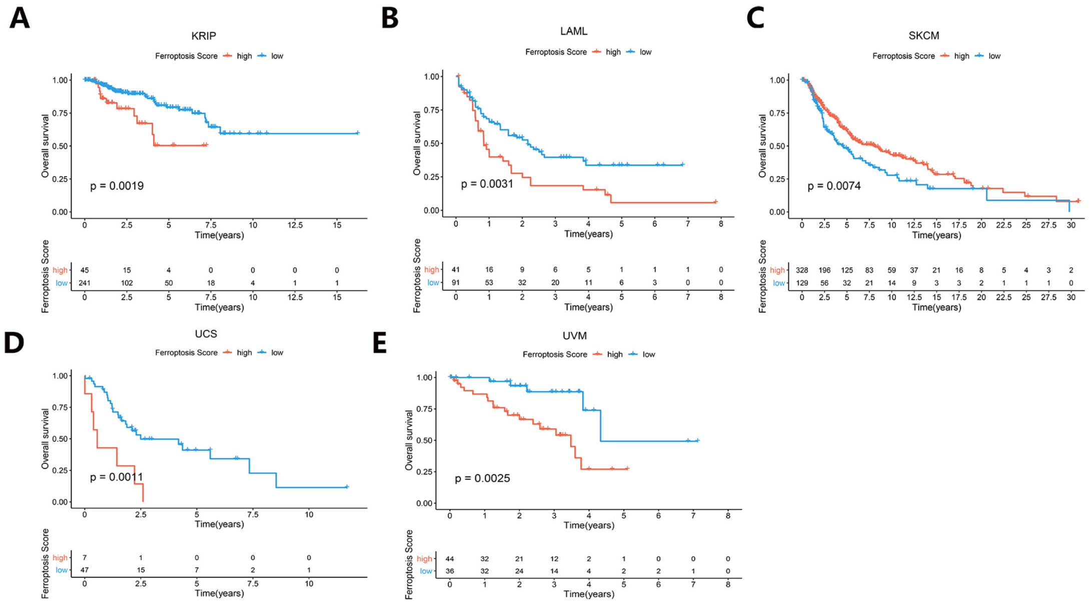

Supplementary Figure 5. Kaplan-Meier survival curves of overall survival (OS)

of high ferroptosis score vs. low ferroptosis score among the cancers. (A-E) There was a positive relationship between ferroptosis score and SKCM, while other cancers showed a negative correlation with ferroptosis score, including KIRP, LAML, UCS and UVM. The P-values calculated by log-rank test are represented.

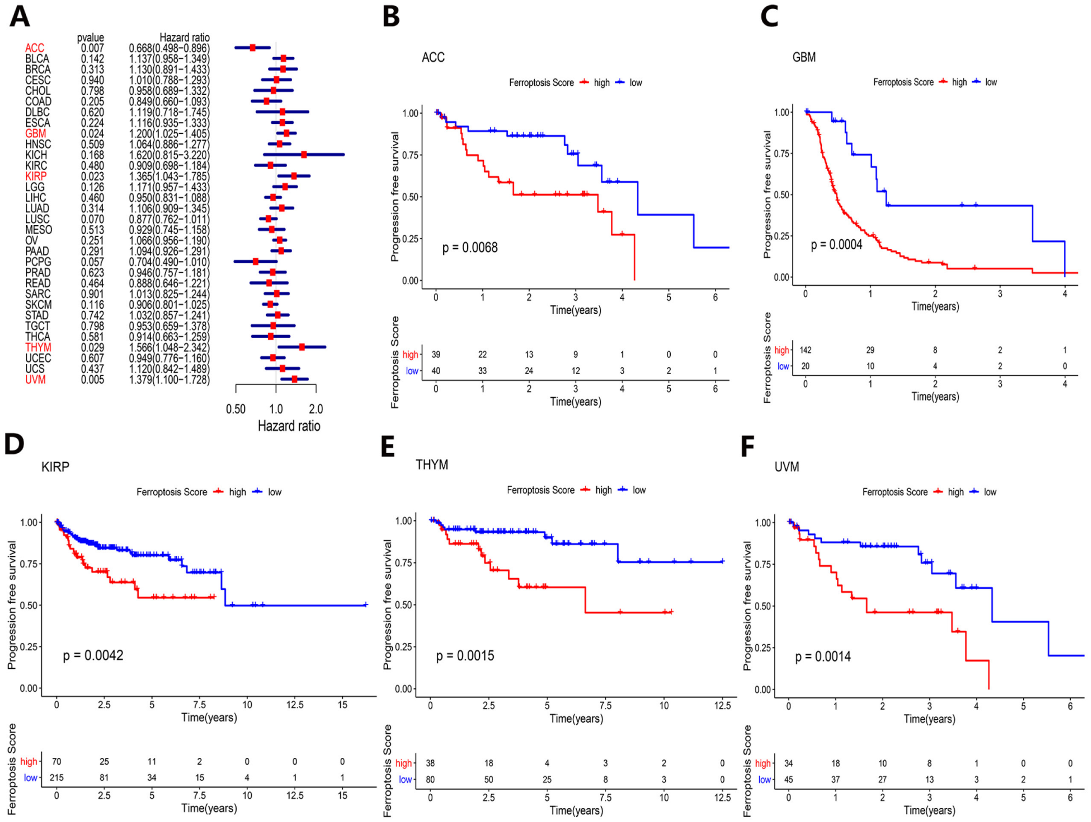

Supplementary Figure 6. Analysis of patient progression-free survival (PFS). (A) The distribution of the hazard ratios of PFS across 33 cancer types. **(B-F)** Kaplan-Meier estimates of progression-free survival of patients with 1 of 5 cancer types and a high ferroptosis score or a low ferroptosis score.

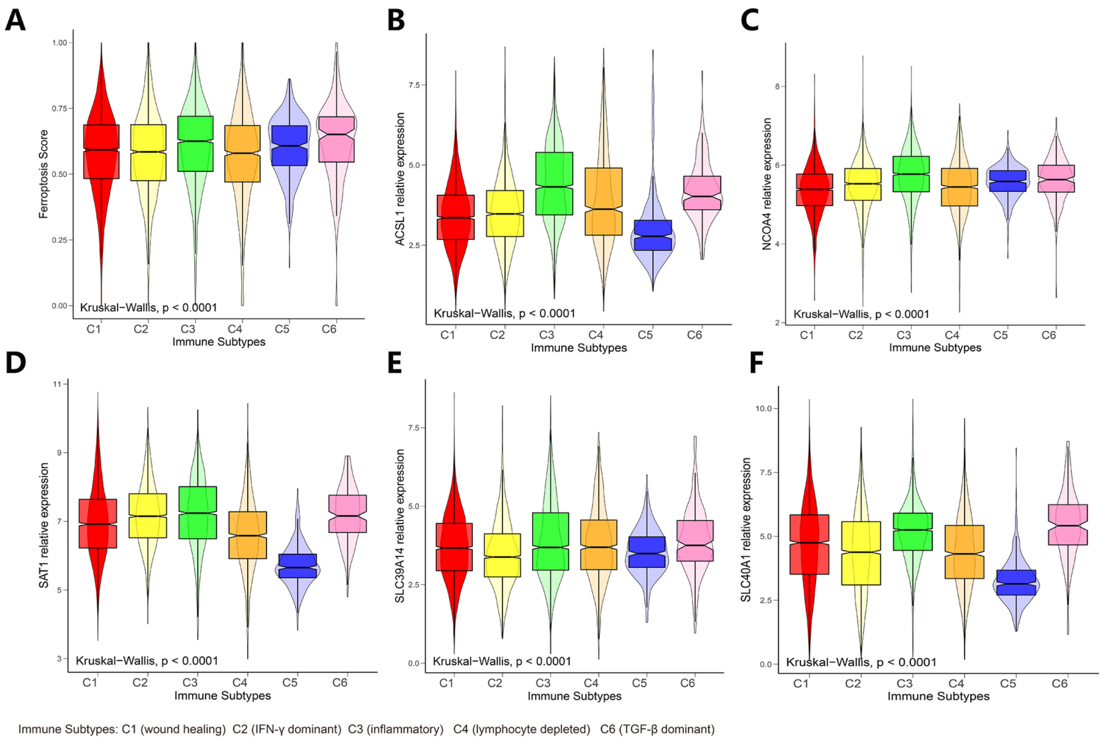

Supplementary Figure 7. Violin diagrams of ferroptosis regulator expression levels in 6 immune cell subtypes.

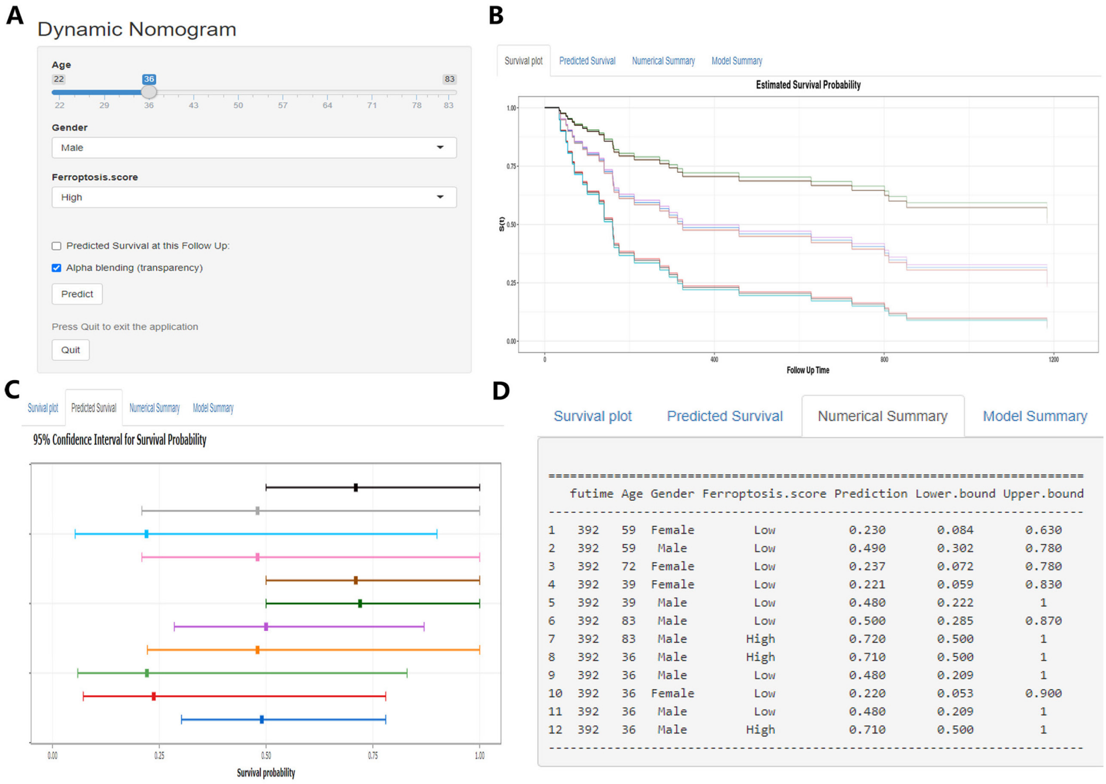

Supplementary Figure 8. Online webserver construction based on the nomogram

to predict survival of immunotherapy cohort on the basis of age, gender and ferroptosis score. (A) Control panel. **(B)** Correspondingly survival plot. **(C)** Survival probability. **(D)** Summary of patients.
